# Morphological and molecular diversity of Lake Baikal candonid ostracods, with description of a new genus

**DOI:** 10.3897/zookeys.684.13249

**Published:** 2017-07-11

**Authors:** Ivana Karanovic, Tatiana Ya. Sitnikova

**Affiliations:** 1 Department of Life Science, College of Natural Science, Hanyang University, Seoul 133–791, South Korea; Institute for Marine and Antarctic Studies, University of Tasmania, Private Bag 49, 7001, Hobart, Tasmania, Australia; 2 Limnological Institute, Siberian Branch, Russian Academy of Sciences, P.O. Box 664033, Irkutsk, Russia

**Keywords:** Crustacea, Deep lakes, molecular phylogeny, taxonomy, CO1, 16S rRNA, 18S rRNA, 28S rRNA

## Abstract

Uncoupling between molecular and morphological evolution is common in many animal and plant lineages. This is especially frequent among groups living in ancient deep lakes, because these ecosystems promote rapid morphological diversification, and has already been demonstrated for Tanganyika cychlid fishes and Baikal amphipods. Ostracods are also very diverse in these ecosystems, with 107 candonid species described so far from Baikal, majority of them in the genera *Candona* Baird, 1845 and *Pseudocandona* Kaufmann, 1900. Here we study their morphological and molecular diversity based on four genes (two nuclear and two mitochondrial), 10 species from the lake, and 28 other species from around the world. The results of our phylogenetic analysis based on a concatenated data set, along with sequence diversity, support only two genetic lineages in the lake and indicate that a majority of the Baikal *Candona* and *Pseudocandona* species should be excluded from these genera. We describe a new genus, *Mazepovacandona* **gen. n.**, to include five Baikal species, all redescribed here. We also amend the diagnosis for the endemic genus *Baicalocandona* Mazepova, 1972 and redescribe two species. Our study confirms an exceptional morphological diversity of Lake Baikal candonids and shows that both Baikal lineages are closely related to *Candona*, but only distantly to *Pseudocandona*.

## Introduction

In the past decade the number of Candonidae genera and species has almost doubled, so that now the family contains about 500 Recent species in 39 genera and eight tribes (see Martens and Savatenalinton 2011; Karanovic 2012). This increase is mostly due to the study of previously poorly sampled regions, such as Australia (Karanovic 2007) and South America (Karanovicand Datry 2009; Higutiand Martens 2012, 2014). Almost each genus described from those regions is supported by numerous synapomorphic characters, with phylogenetically resolved position within the family Candonidae. This has been supported by a cladistic analysis of the family based on morphological characters (Karanovic 2007). In this analysis, Candonini is the only tribe which seems not to be monophyletic. It comprises the greatest number of genera (12), most of which are Holarctic and described more than 100 years ago (Meisch 2000).

There were several attempts to revise some of the most specious and taxonomically problematic Candonini genera, such as *Candona* Baird, 1845, *Fabaeformiscandona* Krstić, 1972, *Pseudocandona* Kaufmann, 1900, and *Typhlocypris* Vejdovský, 1882 (see Meisch 1996; Karanovic 2005, 2006, 2013; Namiotko et al. 2014). However, the results are only partial because the current diagnosis of both *Candona* and *Pseudocandona* exclude almost all Baikal Lake representatives of these two genera.

A majority of 104 Baikal candonids were described in two main publications: Bronstein (1947) and Mazepova (1990). There are three genera in the lake: *Candona* Baird, 1845 (with 48 species and 5 subspecies), *Pseudocandona* Kaufmann, 1900 (27 species and 3 subspecies), and *Baicalocandona* Mazepova, 1976 (11 species and 10 subspecies). Only *Baicalocandona* is endemic. The original descriptions, although missing some important taxonomic information, revealed a great morphological diversity and indicated that Baikal candonids need to be revised and probably subdivided into several genera (Karanovic 2007, 2012; Danielopol et al. 2011). Only two species, *Pseudocandona gajewskajae* Bronstein, 1947 and *P. ceratina* Mazepova, 1982 were studied after their original descriptions (Martens et al. 1992a, b). The authors provided more morphological details of the two species and concluded that their position within *Pseudocandona* is dubious. Similarly, Baikal amphipods are also extremely morphologically diverse, but recent studies showed that a morphologically diverse family Acanthogammaridae is monophyletic, while morphologically conservative Micruropodidae is paraphyletic (Macdonald III et al. 2005). In general, morphological and molecular evolution have been uncoupled not only in ancient lakes (Martens 1994), but also in other ecosystems and across all life kingdoms (Pisani et al. 2007; Renaud et al. 2007; Sotiaux et al. 2009; Poisot et al. 2011; Dávalos et al. 2012).

Lake Baikal is a place of exceptional biodiversity. Over 2500 species have been recorded so far, more than half of them endemic to the lake (Timoshkin 2001). Crustaceans are especially diverse, with amphipods having nearly 300 species (Takhteev 2000). Ostracods are the second most diverse crustacean group with 90% species endemic to the lake (Martens 1994). Besides candonids, Lake Baikal is a biodiversity hot spot for another unrelated ostracod group, Cytheroidea, with almost all species assigned to a single genus, *Cytherissa* Sars, 1925 (47 species and 10 subspecies). Schön and Martens (2012) compared molecular evolution and phylogeny of cytheroid lineages from Lake Baikal and Lake Tanganyika based on two gene markers, *COI* and ITS. While the latter marker failed to resolve phylogenetic relationships in either of the lakes, *COI* did so in Lake Tanganyika, but not in Baikal. The phylogenetic tree of Lake Baikal cytheroids is awash with multifurcations and the authors conclude that the morphological revision of the Baikal cytheroids is necessary.

In order to recover phylogenetic position of the Lake Baikal candonids within the family we used 10 species from the lake and another 28 from around the word, targeting type species of the genera *Candona, Pseudocandona* and *Fabaeformiscandona*, since the majority of the Baikal species currently belong to the former two genera, and all three genera are also currently polyphyletic (Karanovic 2007; Danielopol et al. 2011). Two nuclear (18S, 28S), and two mitochondrial (16S and *COI*) regions were amplified and a phylogenic tree based on concatenated data set of three genes (two nuclear and 16S) was reconstructed. At the moment, description of all Baikal candonids is not up to the modern standards of ostracod taxonomy and species need to be redescribed. Redescriptions are also necessary in order to provide enough morphological data which can be accurately compared with the level of molecular divergence.

## Material and methods

### Collecting and taxonomy

Samples were taken from 11–15 m depths by SCUBA diving from the shore of Lake Baikal. Three bottom types were sampled: rock, mud, and sand. Ostracods were sorted alive on the spot and immediately fixed in 97% ethyl alcohol. Dissection and identification was done with the aid of Zeiss Axiostar-plus light microscope and Leica DM 2500 compound microscope, equipped with N-Plan objectives, respectively. Scanning Electron Microscope (SEM) photographs were taken with a Hitachi S-4700 at Eulji University (Seoul). Photographs of Zenker organ and hemipenis were taken with Olympus C-5050 digital camera mounted on Olympus PX51 compound microscope.

Collected ostracods were identified with the aid of Mazepova (1990). The terminology for A1, Md, Mxl, L5 and L6 follows Broodbakker and Danielopol (1982), and for L7 Meisch (1996). Here, the view of Meisch (2007) regarding the terminology and homology of the most posterior appendage on the ostracod body (“furca”) is accepted.

### DNA extraction and amplification

In the first step of the DNA extraction specimens were kept for 2–3 hours in distilled water. LaboPass Tissue Mini extraction kit (Cosmo Genetech Co., LTD, Korea) was used in all further steps of extraction, following the manufacturer's protocol. Fragments of *COI* were amplified using universal Folmer primers (Folmer et al. 1994). Fragments of 28S were amplified using the primer pairs dd/ff, ee/mm, vv/xx from Hillis and Dixon (1991), of the 18S with primers from Yamaguchi (2003), and fragments of 16S were amplified with primers from Palumbi et al. (1996), all using a TaKaRa PCR Thermal Cycler Dice. For all amplifications PCR reactions were carried out in 25 μl volumes, containing: 5 μl of DNA template, 2.5 μl of 10× ExTaq Buffer, 0.25 μl of TaKaRa Ex Taq (5 units/ μl), 2 μl of dNDTP Mixture (2.5 mM each), 1 μl each primer, and 13.25 μl distilled H2O. The PCR protocol for *COI* amplification consisted of initial denaturation for 5 minutes at 94°C, 40 cycles of denaturation for 1 minute at 94°C, annealing for 2 minutes at 46°C, extension for 3 minutes at 72°C, and final extension at 72°C for 10 minutes. Protocol for 28S consisted of initial denaturation for 5 minutes at 94°C, 40 cycles of denaturation for 35s at 95°C, annealing for 1 minute at 50°C, extension for 1 minute at 72°C, and final extension at 72°C for 5 minutes. PCR settings for the amplification of 18S followed Yamaguchi (2003) for each corresponding primer pair. Settings for 16S consisted of initial denaturation at 94°C for 5 minutes, 35 cycles of denaturation for 30s at 94°C, annealing for 30s at 48°C, extension for 1 minute at 72°C, and final extension at 72°C for 10 minutes. The PCR products were electrophoresed on 1% agarose gels; if DNA was present the products were purified for sequencing reactions using the LaboPass PCR Purification Kit, following the guidelines provided with the kit. DNA was sequenced on an ABI automatic capillary sequencer (Macrogene, Seoul, South Korea) using the same set of primers always in both directions.

### Molecular data analysis

All sequences were visualized using Finch TV version 1.4.0 (http://www.geospiza.com/Products/finchtv.shtml). BLAST (Altschul et al. 1990) analysis of GenBank database were used to check that the obtained sequences were ostracod in origin and not contaminants. Each sequence was checked for the quality of signal and sites with possible low resolution, and corrected by comparing forward and reverse strands. Sequences were aligned in MEGA 7 (Kumar et al. 2016) with ClustalW (Thompson et al. 1994) with extension penalty changed from default settings (6) to 1 for 28S dataset in order to allow alignment of homologous regions that were separated by expansion segments present in some taxa but not others. All alignments were manually checked and corrected where necessary. The 28S alignments were also checked with Gblock (Castresana 2000) and ambiguous blocks were removed. We performed analyses of the concatenated dataset including 18S, 28S, and 16S fragments. Datasets for some species were composed of sequences acquired from different specimens in order to avoid missing data, and for our outgroup we combined 16S from two different, but closely related, species. All specimens of one species came from the same locality and their identity was confirmed by close morphological examination. Missing data were coded “?". Recent simulations and empirical analyses suggested that missing data in Bayesian phylogenetics are not themselves problematic, and that incomplete taxa can be accurately placed as long as the overall numbers of characters are large (Wiens 2003; Wiens and Moen 2008). Sequence differences were calculated in MEGA 7 using uncorrected p-distance method. For the best fit evolutionary model program jModelTest 2.1.6 (Darriba et al. 2012; Guindon and Gascuel 2003) was used with the Akaike information criterion (Hurvich and Tsai 1989). Bayesian inference reconstruction in MrBayes v3.2.6 (Huelsenbeck and Ronquist 2001; Ronquist and Huelsenbeck 2003; Ronquist et al. 2012) was performed with the best fit model and priors for the base and state frequencies calculated by jModelTest. Data were partitioned into five blocks corresponding to gene regions, each with its fixed priors. The 28S data set was analyzed as three independent fragments: d/f; e/m, and v/x, corresponding to the primer sets used for their amplification. All analyses ran with four chains simultaneously for two million generations in two independent runs, sampling trees every 200 generations. Of the four chains three were heated and one was cold, the temperature value (“Temp” command in MrBayes) was 0.1 (default option). The results were summarized and trees from each MrBayes run were combined with the default 25% burn-in. A >50% posterior probability consensus tree was constructed from the remaining trees. For the choice of the outgroup we relied on the phylogeny published in Hiruta et al. (2016). Since the relationships within Cypridoidea was not clearly resolved and Candonidae appears as a sister taxon to all other Cypridoidea, we decided on a representative of Cyclocyprididae, which used to be in the same family with Candoninae. Sequence GenBank accession numbers are listed in Supplement 1. Software FigTree v1.4.3 was used for tree visualizations.

## Results

### Taxonomy

#### Genus Mazepovacandona gen. n.

Taxon classificationAnimaliaPodocopidaCandonidae

http://zoobank.org/3CEDBD01-E93F-499C-991A-984D6B089700

##### Type species.

*Mazepovacandona directa* (Bronstein, 1947), comb. n.

##### Other species.

*M. godlewski* (Mazepova, 1984), comb.n., *M. navitarum* (Mazepova, 1976), comb. n., *M. orbiculata* (Mazepova, 1990), comb. n., *M. spicata* (Mazepova, 1982), comb. n.

##### Diagnosis.

Shell shape variable, but surface generally smooth or poorly ornamented. A1 7- or 6-segmented. Male A2 with t-setae transformed into sensory setae, z-setae transformed into claws. Female A2 G2-claw considerably shorter than G1 or G3. Exopod of A2 consisting of small plate and three setae of which one is long. Male prehensile palps asymmetrical and both with hook-like fingers. L6 with basal seta and with one seta on each endopodal segment, except on last segment, which carries two setae and one claw. L7 with only d1- and dp-setae on basal segment, e- and f-setae missing, g-seta long; terminal segment with short h1-seta and h2- and h3-setae equally long; penultimate segment divided or incompletely divided. UR with both claws and setae present. Zenker organ with variable number of spine whorls, varying from 3+2 to 5+2; anterior part (cap) hemispherical, lattice-like structure well-developed. Hemipenis with small a-lobe not projecting laterally; M-peace terminally rounded (ball-like); ejaculatory process (bursa copulatrix) terminally pointed.

##### Etymology.

The genus is named after late Dr. Galina Mazepova as an acknowledgment of her outstanding contribution to the study of Lake Baikal ostracod fauna.

##### Remarks.

*Mazepovacandona* currently contains five morphologically diverse species. The carapace shape (from triangular to banana shaped) is only one example of this diversity. The number of segments on the antennule and the way male z-setae on the second antenna are developed is also variable, however all females have G2-claw on the second antenna shorter than the rest of the claws. The number of setae on the second segment of the Md-palp is also variable and it can be either three or four. Prehensile palps are dissimilar among species, although all have clearly pronounced hooked-like fingers on both left and right palp. The basal seta (d1) on the walking leg is shorter in all five species than in two *Baicalocandona* species redescribed here. The length of this seta relative to the d2-seta (always absent in Candonidae) is an important taxonomic character in some Cyprididae, such as Cyprinotinae (see the key in Karanovic 2012) and Eucypridinae (see Martens 1989). The d1-seta is often absent in Candonidae, and the importance of its length for the taxonomy of the family has never been studied. In all *Mazepovacandona* the penultimate segment of the cleaning leg is at least partially subdivided, but this tends to be a variable character, for example in *Candona*, *Fabaeformiscandona* (see Meisch 2000), and a few genera from Australia (see Karanovic 2007). The hemipenis morphology in *Mazepovacandona* is characterized by a rounded distal end of the M-peace. The morphology of this part is an important taxonomic character in *Candona* (see Petkovski 1960). Also, the ejaculatory process (*bursa copulatrix*) is pointed in all species of the new genus, but the morphology of this part has not been studied for its taxonomic importance. The hemipenis of the two examined species (*M. directa* and *M. orbiculata*) was in an erected state and because of that the position of the a-lobe and its shape were not easy to observe. It is interesting to note that all examined males of *M. directa* had their hemipenis erected. The hemipenis illustrations of these two species in Mazepova (1990) also show an erected copulatory organ. The Zenker organ has a balloon-like anterior end, a characteristic which has been noted in *Pseudocandona inaequivalvis baikalensis* Bronstein, 1947, some *Undulacandona* species (see Smith 2011; Karanovicand Cho 2017), and in the families Cyclocyprididae and Paracyprididae (see Danielopol 1982). The morphology and development of the Zenker organ has been studied recently (see Yamada and Matzke-Karasz 2012; Yamada et al. 2014). The phylogenetic importance of its morphology is recognized on the higher taxonomic levels (Danielopol 1978; Matzke-Karasz 1997), but not well understood at the generic or even family level. Many of the Candonidae genera have the number of whorls of spines as a part of their diagnosis. In the new genus, the number ranges from five to seven, and they all have well-developed spines, which is a sign of the sexual maturity (Yamada et al. 2014). Interestingly, Kesling (1957) reported a variability of the whorl numbers in one *Candona* species, where some individuals have seven and others eight whorls. The latter number is very unusual in the family Candonidae, where the number of whorls never exceeds seven.

Despite the morphological diversity of *Mazepovacandona*, this genus seems to be most closely related to *Candona* and *Fabaeformiscandona*. For example, prehensile palps of *M. directa* (elongated) are very similar to *candida*-group of *Candona*, while female genital lobe bears similarity to the *neglecta*-group. There is also similarity with *Fabaeformiscandona*, especially because several of its species have rounded distal part of the M-peace. The *breuli*-group of the latter genus is particularly similar to *Mazepovacandona* in sense that the M-peace is not so strongly sclerified and that most species have an UR with a long posterior seta. However, most of the species currently belonging to this group have a completely fused penultimate segment of the cleaning leg.

#### Mazepovacandona directa

Taxon classificationAnimaliaPodocopidaCandonidae

(Bronstein, 1947)

**Figure 1.**
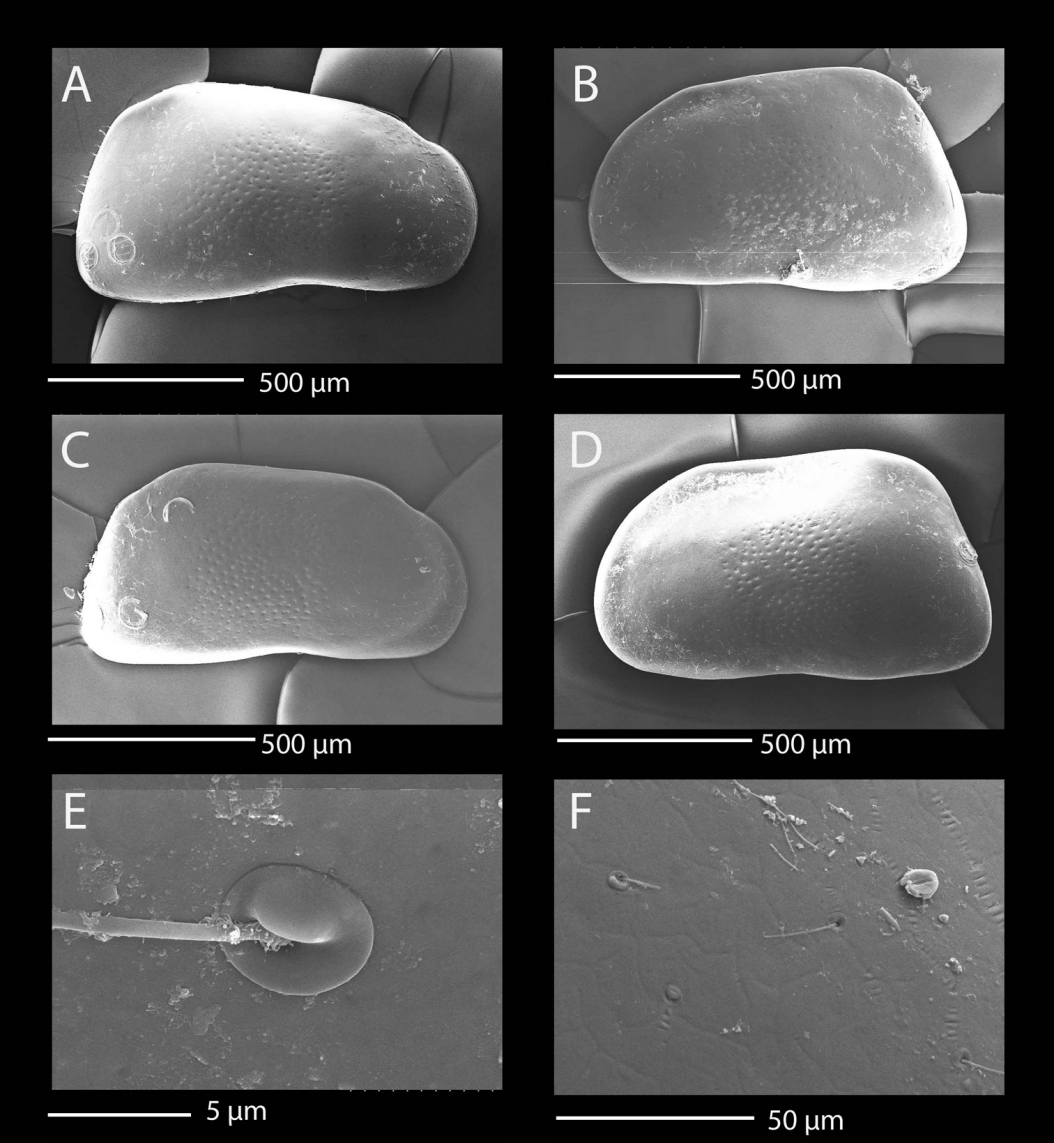
SEM photographs of *Mazepovacandona directa* (Bronstein, 1947). **A, B, E, F** male **C, D** female: **A** RV, lateral view **B** LV, lateral view **C** RV, lateral view **D** LV, lateral view **E** detail of a sensilla **F** detail of the shell surface.

**Figure 2.**
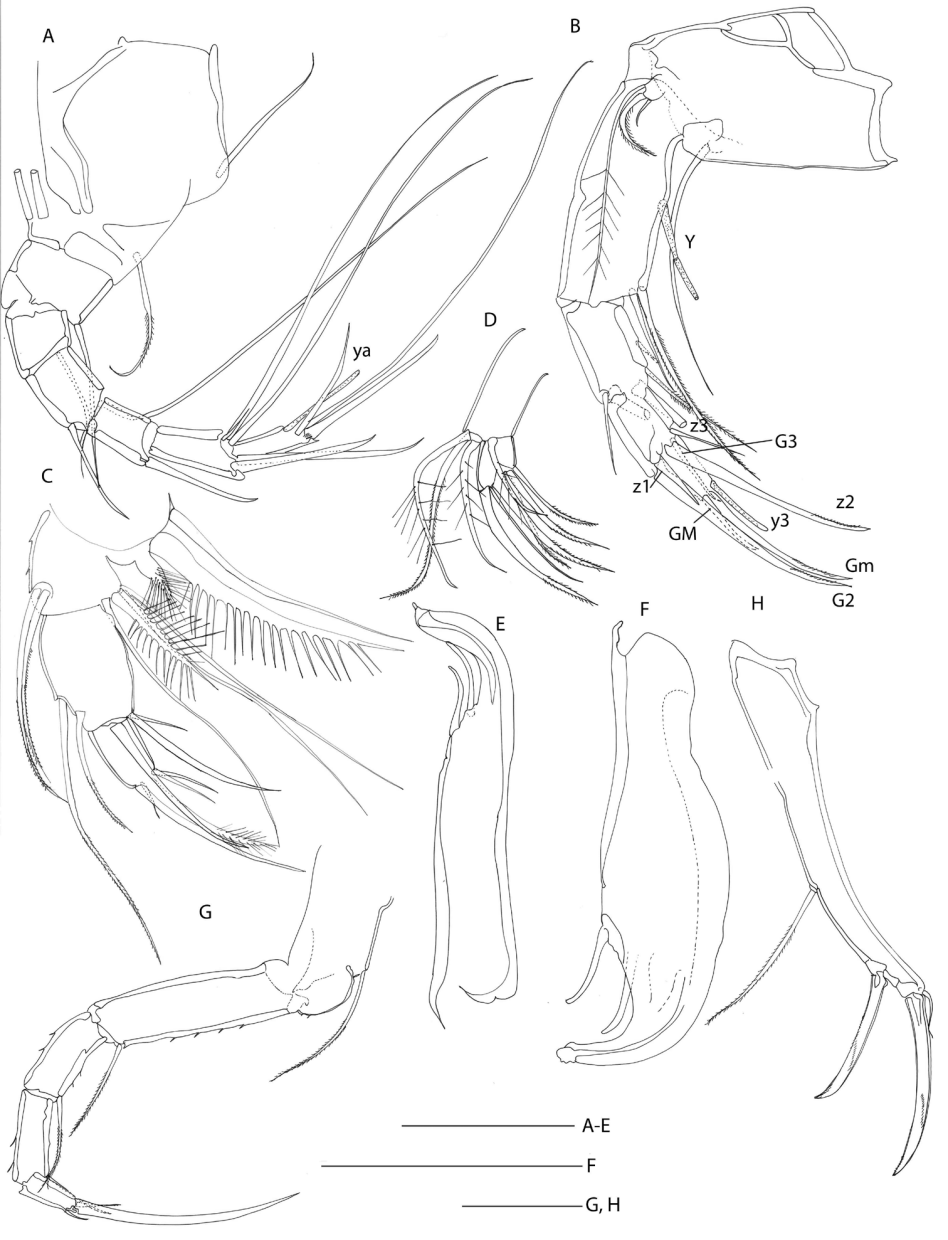
Line drawings of *Mazepovacandona directa* (Bronstein, 1947). Male. **A** A1 **B** A2 **C** Md-palp **D** Mxl-palp **E, F** prehensile palps **G** L6 **H** UR. Scales = 0.1 mm.

**Figure 3.**
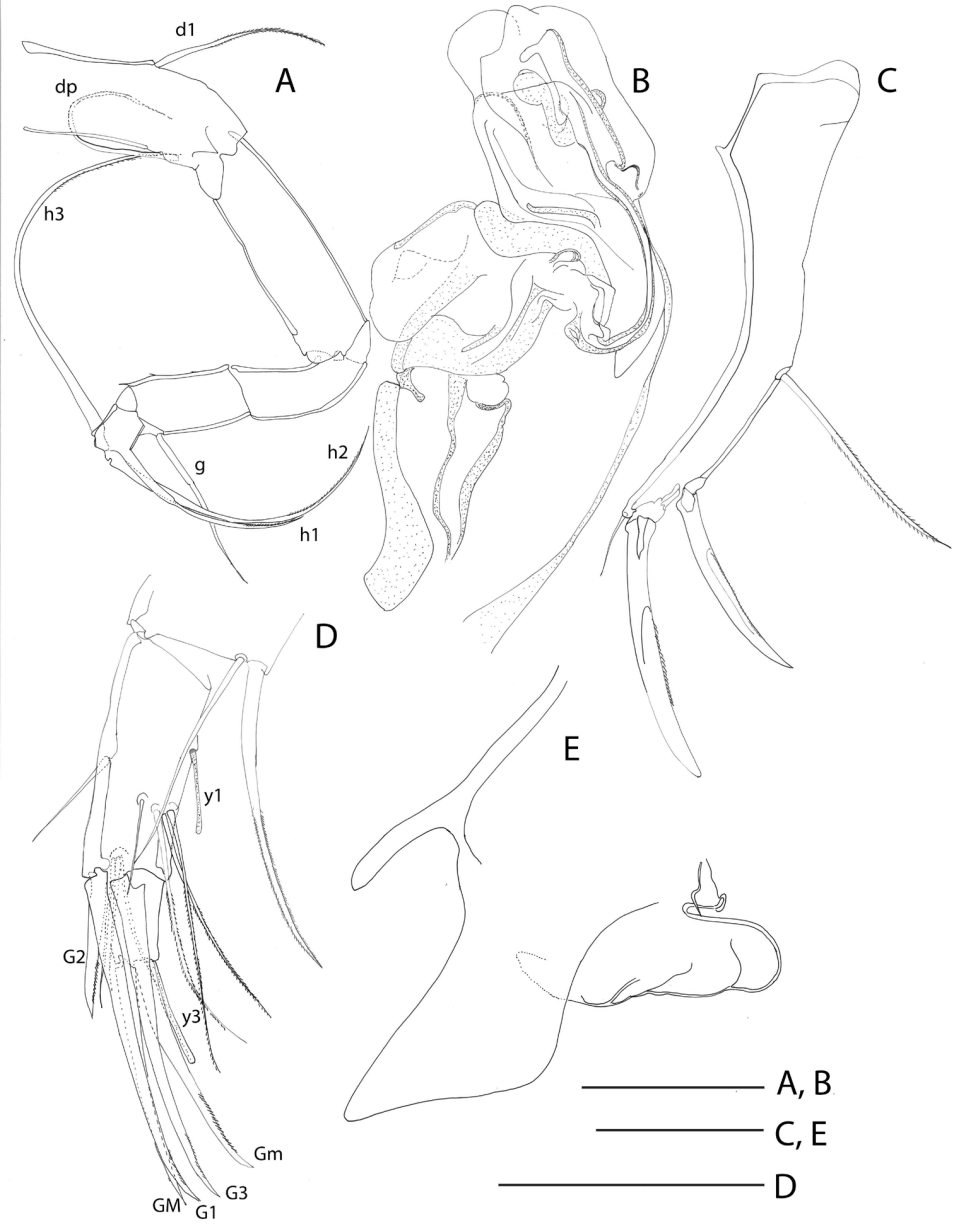
Line drawings of *Mazepovacandona directa* (Bronstein, 1947). **A, B** male **C–E** female **A** L7 **B** hemipenis **C** UR **D** A2 **E** genital segment. scales = 0.1 mm.

Figs 1 , 2 , 3 , 14D , 15D

Candona directa sp. n. – Bronstein (1947): p.12, fig. 121
Candona directa Bronstein – Mazepova (1990): p. 73, fig. 20B

##### Material examined.

Two males and one female dissected and mounted on glass slides (shell of one male and one female on one SEM stub), 25 undissected specimens in 95% alcohol, 1 specimen used for the DNA extraction, all collected from 12–15 m depth by SCUBA diving off Listvyanka, 51°51'51.3"N, 104°50'37.8"E, 12 September 2015, collectors: Igor Khanaev and Ivan Nebesnykh.

##### Short redescription.

Almost no sexual dimorphism in shell shape in lateral view (Fig. 1A–D). Both LV and RV subrectangular, dorsal margin straight and strongly inclined towards anterior end. Posterior end straight, anterior end rounded. RV with small recess antero-dorsally, ventral margin very slightly concave. Surface only centrally ornamented with shallow pits. Surface cuticular pores of two types: with simple lip and with semi operculum (Fig. 1E, F). Length around 1 mm.

A1 7-segmented with posterior setae transformed into claws (Fig. 3A). Male A2 with subdivided penultimate segment and t2- and t3-setae transformed into sexual bristles; z1- and z2-setae transformed into claws, as well asz3-seta; G1- and G3-claws reduced and short, G2-claw long (Fig. 2B). Female A2 (Fig. 3D) with all three z-seta untransformed; G2 claw short, and only slightly exceeding distal margin of terminal segment. Md-palp (Fig. 2C) with 3+2 setae on inner side, gamma seta not plumose. Mxl-palp (Fig. 2D) with rectangular terminal segment. Prehensile palps (Fig. 2G, H) with long bodies and short, curved fingers. L6 (Fig. 2G) with basal seta reaching far beyond distal margin of basal segment. L7 (Fig. 3A) clearly 5-segmented; basal segment with d1- and dp-seta; no e- or f-setae, g-seta long; terminal segment with setae h2- and h3- long and h1-seta shorter, but also considerably long. UR in both sexes (Figs 2H, 3C) very similar and robust, with long posterior seta and strong claws. Hemipenis always (?) in semi-erectile mode (Fig. 3B, 14D); a-lobe relatively small but its shape hard to accurately perceive due to its folded position; M-peace rounded distally, ejaculatory tube with pointed distal end. Female genital field (Fig. 3E) with enlarged, semi-triangular lobe. Zenker organ with 5+2 whorls of spines (Fig. 15D); anterior cap hemispherical, with strongly sclerified margin, lattice very elaborately developed and on side adjoined by longer spines. Eyes present, white.

#### Mazepovacandona godlewski

Taxon classificationAnimaliaPodocopidaCandonidae

(Mazepova, 1984)

Figs 4 , 5

Candona godlewski sp. n. – Mazepova (1984): p. 38, fig. 12 (1–10)
Candona godlewski Mazepova – Mazepova (1990): p. 43, fig. 4, 5B.

##### Material examined.

One females dissected and mounted on glass slides (shell on the SEM stub), 2 undissected specimens in 95% alcohol, 2 specimens used for the DNA extraction), all collected from 12–15 m depth by SCUBA diving off Listvyanka, 51°51'51.3"N, 104°50'37.8"E, 12 September 2015, collectors: Igor Khanaev and Ivan Nebesnykh.

##### Short redescription.

**Figure 4.**
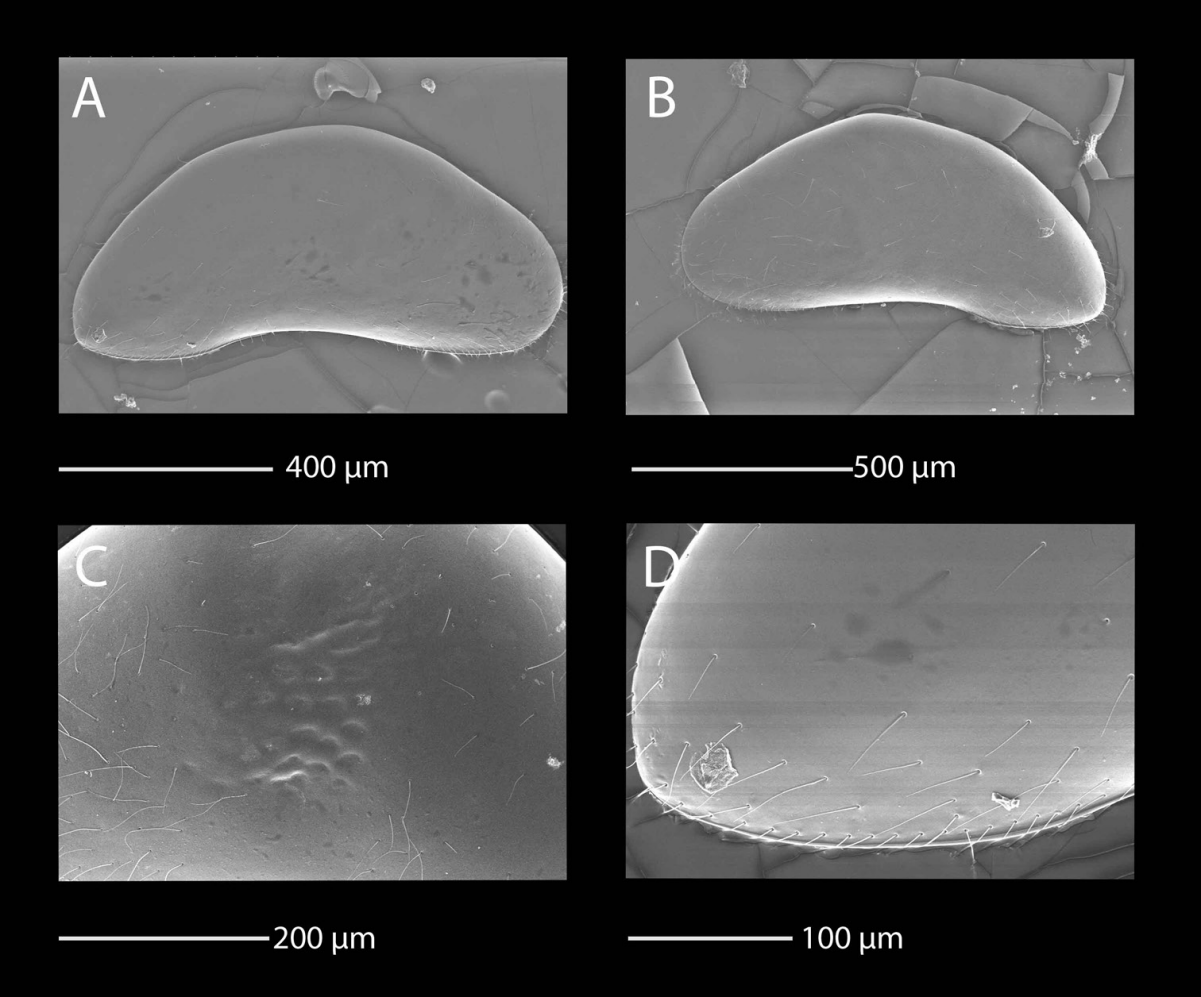
SEM photographs of *Mazepovacandona godlewski* (Mazepova, 1984). Female. **A** RV, lateral view **B** LV, lateral view **C** details of the fine surface ornamentation **D** details of the posterior end of the RV.

Both LV and RV banana shaped (Fig. 4A, B) with dorsal margin rounded, strongly arched and narrow ends, posterior end narrower than the anterior one. Valve margins framed with narrow fringe. Surface smooth, with few shallow pits only centrally (Fig. 4C). Surface cuticular pores simple, without lip (Fig. 4D). Length around 1 mm.

**Figure 5.**
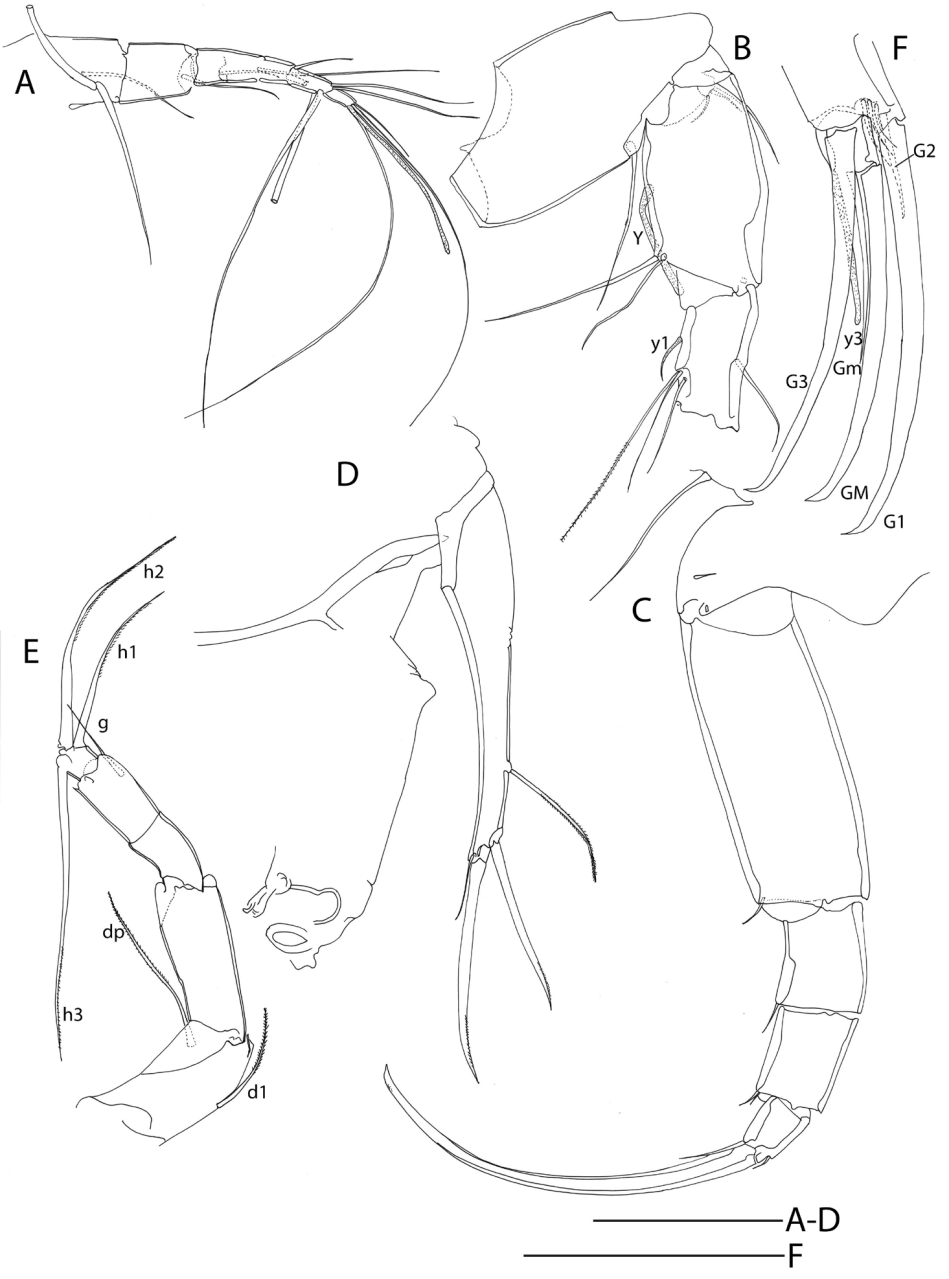
Line drawings of *Mazepovacandona godlewski* (Mazepova, 1984). Female. **A** A1 **B** First three segments of A2 **C** L6 **D** UR **E** L7 **F** penultimate and terminal segments of A2. Scales = 0.1 mm.

A1 7-segmented, with segments 3 and 4 partly fused (Fig. 5A), posterior setae thin. Male A2 with subdivided penultimate segment and t2- and t3-setae transformed into sexual bristles. Female A2 (Fig. 5B, F) with all three z-seta untransformed; G2 claw short, and only slightly exceeding margin of the terminal segment. Md-palp with 4+2 setae on the inner side, gamma seta not plumose. L6 (Fig. 5C) with basal seta reaching beyond basal segment. L7 (Fig. 5E) clearly 5-segmented; basal segment with d1- and dp-seta; no e-or f-setae, g-seta long; terminal segment with setae h2- and h3- long and h1-seta shorter, but also considerably long. UR (Fig. 5D) with long posterior seta, genital field with small semi-triangular lobe. Eyes large and dark.

Females not collected.

#### Mazepovacandona navitarum

Taxon classificationAnimaliaPodocopidaCandonidae

(Mazepova, 1976)

**Figure 6.**
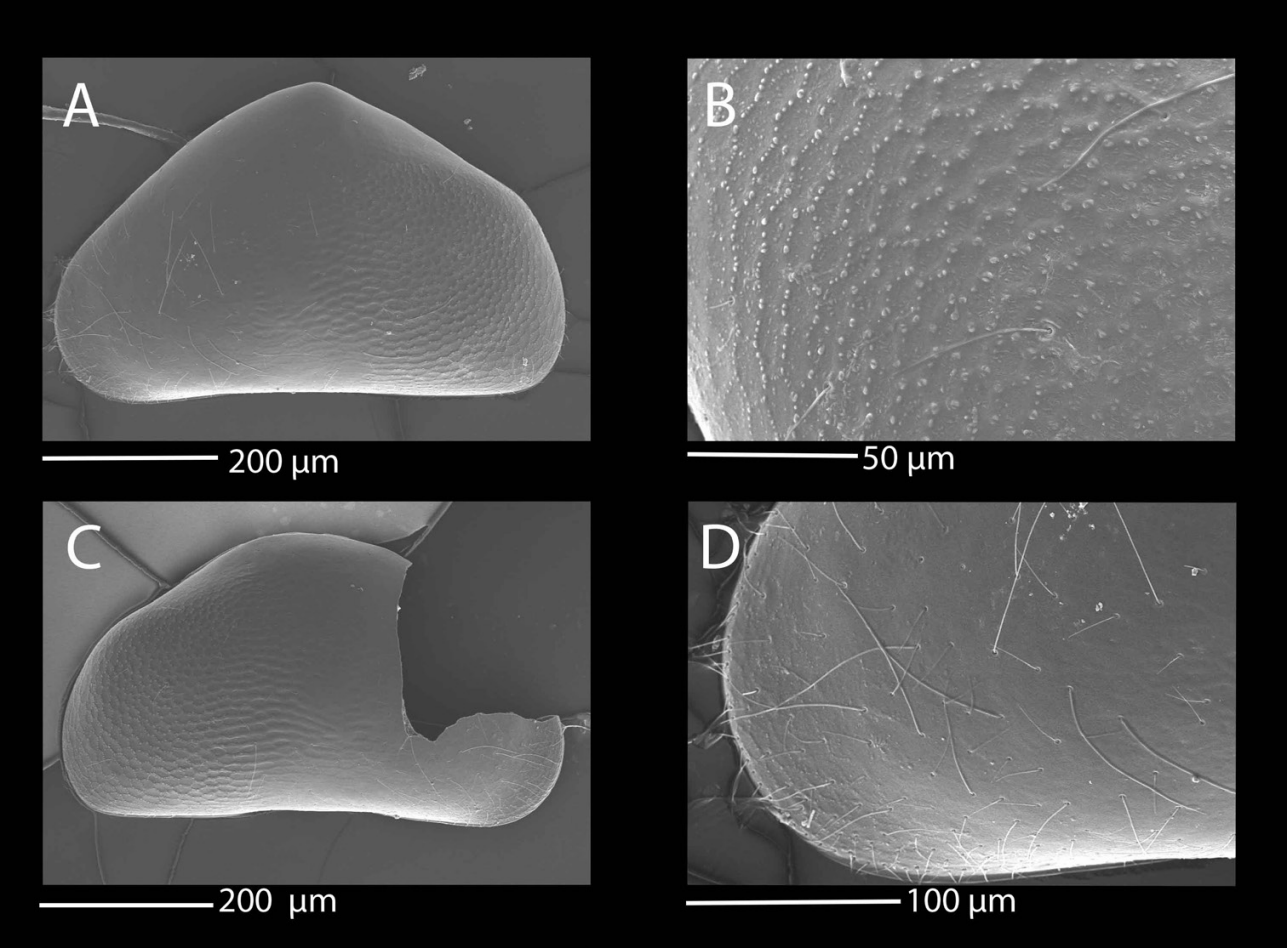
SEM photographs of *Mazepovacandona navitarum* (Mazepova, 1976). Male. **A** LV, lateral view **B** details of the surface ornamentation **C** RV, lateral view **D** details of the anterior end LV.

**Figure 7.**
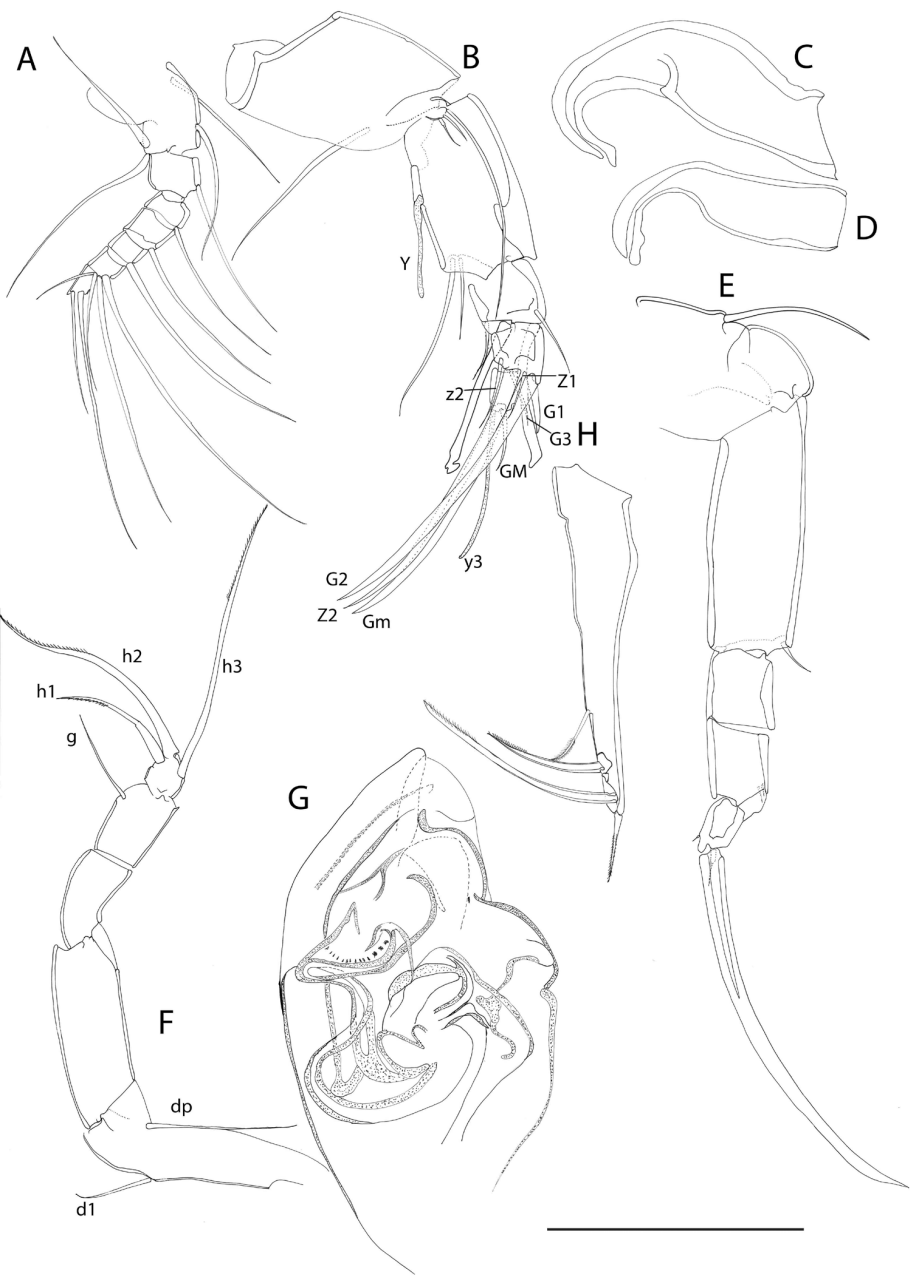
Line drawings *Mazepovacandona navitarum* (Mazepova, 1976). Male. **A** A1 **B** A2 **C, D** prehensile palps E L6 **F** L7 **G** hemipenis **H** UR. Scale = 0.1 mm.

Figs 6 , 7 , 14C , 15C

Baicalocandona navitarum sp. n. – Mazepova (1976): p. 72, fig. 9
Baicalocandona navitarum Mazepova – Mazepova (1990): p. 292, fig. 125, 126B

##### Material examined.

One male soft body used for DNA extraction and after that dissected and mounted on a glass slide (shell of one SEM stub), collected from 12–15 m depth by SCUBA diving off Listvyanka, 51°51'51.3"N, 104°50'37.8"E, 12 September 2015, collectors: Igor Khanaev and Ivan Nebesnykh.

##### Short redescription.

Valves asymmetrical: LV subtriangular with pointed dorsal margin, RV with rounded dorsal margin (Fig. 6A, C). Posterior end much narrower than anterior end. Surface with shallow pits and reticulation only on the anterior part of the shell. Surface cuticular pores of only one type, with small lip (Fig. 6B, D). Size about 0.6 mm.

A1 7-segmented. Male A2 with subdivided penultimate segment and t2- and t3-setae transformed into sexual bristles; z2-setae transformed into claw, z1- and z3-setae untransformed; G1- and G3-claws reduced and short, G2-claw long (Fig. 7B). Prehensile palps (Fig. 7C, D) with almost equally long bodies and fingers, fingers hook-shape. L6 (Fig. 7E) with basal seta reaching far beyond distal margin of basal segment. L7 (Fig. 7F) clearly 5-segmented; basal segment with d1- and dp-setae; no e- or f-setae, g-seta long; terminal segment with setae h2- and h3- long and h1-seta shorter. UR (Fig. 7H) with all setae and claws; posterior seta not particularly long, and posterior claw almost half as long as anterior one. Hemipenis (Figs 7B, 14C) with a-lobe relatively triangular and not projecting; M-peace rounded distally, ejaculatory tube with pointed distal end. Zenker organ (Fig. 15C) with 3+2 whorls of spines; anterior cap hemispherical, with strongly sclerified margin.

Females not collected.

#### Mazepovacandona orbiculata

Taxon classificationAnimaliaPodocopidaCandonidae

(Mazepova, 1990)

**Figure 8.**
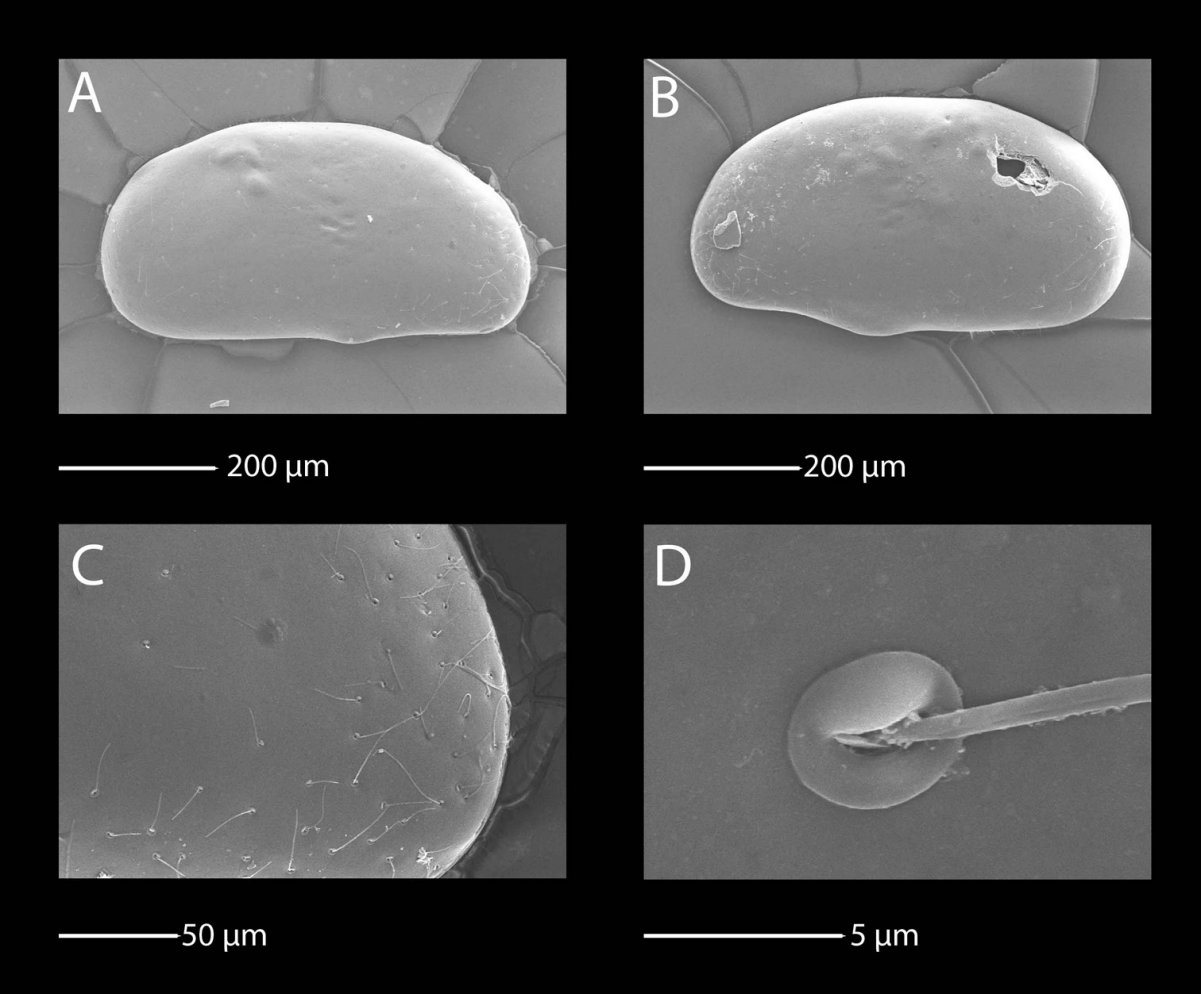
SEM photographs of *Mazepovacandona orbiculata* (Mazepova, 1990). Male. **A** RV, lateral view **B** LV, lateral view **C** details of the anterior end RV **D** detail of the surface sensilla.

**Figure 9.**
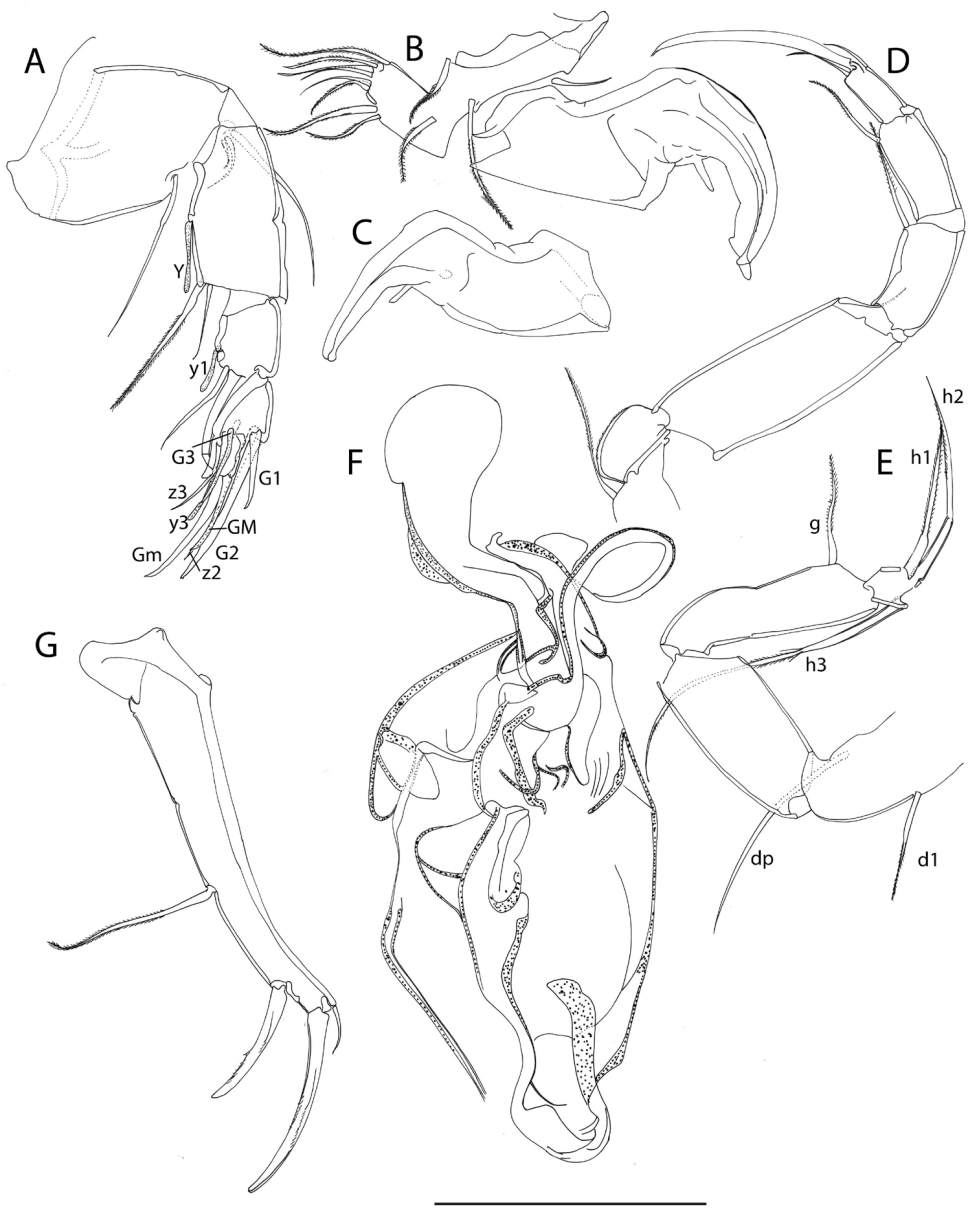
Line drawings *Mazepovacandona orbiculata* (Mazepova, 1990). Male. **A** A2 **B, C** prehensile palps **D** L6 **E** L7 **F** hemipenis **G** UR. Scale = 0.1 mm.

Figs 8 , 9 , 14E , 15E

Candona orbiculata sp. n. – Mazepova (1990): p. 95, fig. 30

##### Material examined.

One male soft body used for DNA extraction and after that dissected and mounted on one glass slide (shell on SEM stub), three juveniles kept in 95% alcohol, all collected from 12–15 m depth by SCUBA diving off Listvyanka, 51°51'51.3"N, 104°50'37.8"E, 12 September 2015, collectors: Igor Khanaev and Ivan Nebesnykh.

##### Short redescription.

Valves reniform in lateral view, with almost evenly rounded dorsal margin (Fig. 8A, B). Anterior and posterior margins broadly rounded, posterior wider than anterior. Ventral margin almost straight, with bulging around mouth region. Surface smooth and covered with pores, all equipped with semi operculum (Fig. 8C, D). Length around 0.6 mm.

A1 lost during DNA extraction. Male A2 with subdivided penultimate segment and t2- and t3-setae transformed into sexual bristles; z1-seta transformed into claw, z2-setae not observed, z3-seta untransformed; G1- and G3-claws reduced and short, G2-claw long (Fig. 9A). Prehensile palps (Fig. 9B, C) with body and fingers equally long, fingers curved, hook-like. L6 (Fig. 9D) with basal seta reaching far beyond basal segment. L7 (Fig. 9E) not clearly 5-segmented, penultimate segment partially subdivided; basal segment with d1- and dp-setae; no e- or f-setae, g-seta long; terminal segment with setae h2- and h3- long and h1-seta shorter, but also considerably long. UR (Fig. 9G) robust, with long posterior seta and strong claws. Hemipenis always (?) in semi-erectile mode (Fig. 9F, 14E); a-lobe relatively small but its shape hard to accurately perceive due to its folded position; M-peace rounded distally, ejaculatory tube with pointed distal end. Zenker organ (Fig. 15E) with 4+2 whorls of spines; anterior cap hemispherical, with strongly sclerified margin, lattice elaborate, on anterior side adjoined by longer spines. Eyes large and dark.

#### Mazepovacandona spicata

Taxon classificationAnimaliaPodocopidaCandonidae

(Mazepova, 1982)

**Figure 10.**
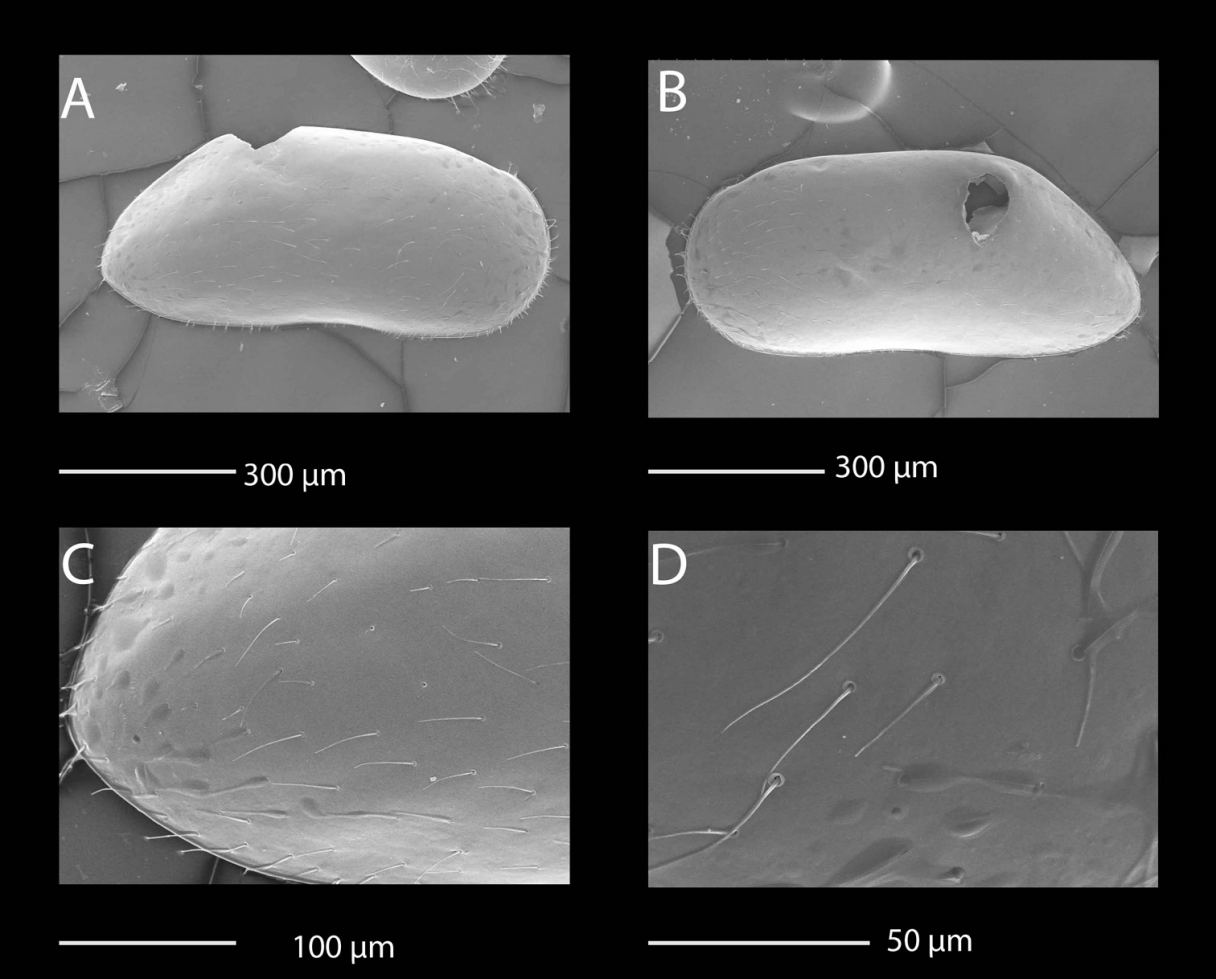
SEM photographs of *Mazepovacandona spicata* (Mazepova, 1982). Male. **A** RV, lateral view **B** LV, lateral view **C** details of the posterior end RV **D** details of the shell surface.

**Figure 11.**
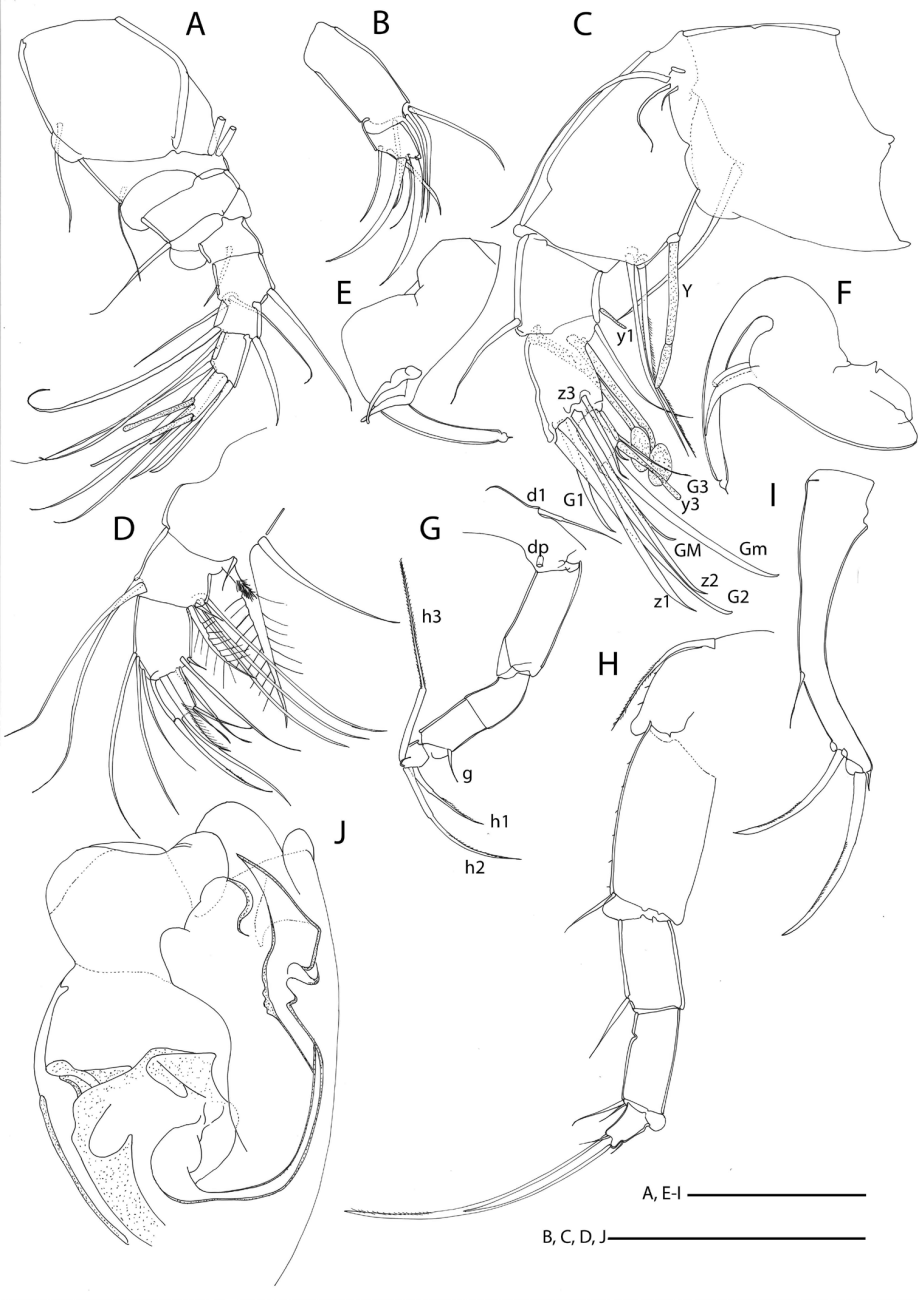
Line drawings of *Mazepovacandona spicata* (Mazepova, 1982). **A** A1 **B** Mxl-palp **C** A2 **D** Md-palp **E, F** prehensile palps **G** L7 **H** L6 **I** UR **J** hemipenis. Scales = 0. 1 mm.

Figs 10 , 11 , 14F , 15F

Candona spicata sp. n. – Mazepova (1982): p. 125, fig. 9A–N
Candona spicata Mazepova – Mazepova (1990): p. 99, fig. 32, 33B

##### Material examined.

One male soft body used for DNA extraction and after that dissected and mounted on one glass slide (shell of one SEM stub), one juvenile kept in 95% alcohol, all collected from 12–15 m depth by SCUBA diving off Listvyanka, 51°51'51.3"N, 104°50'37.8"E, 12 September 2015, collectors: Igor Khanaev and Ivan Nebesnykh.

##### Short redescription.

Valves elongated in lateral view, with almost straight dorsal margin (Fig. 10A, B). Anterior end broadly rounded, posterior margin narrow and inclined. Valve margins framed with narrow fringe (Fig. 10C). Ventral margin concave around middle. Surface smooth and covered with pores, all equipped with lip (Fig. 10D). Length around 0.8 mm.

A1 7-segmented, some posterior setae transformed into claws (Fig. 11A). Male A2 with subdivided penultimate segment and t2- and t3-setae transformed into sexual bristles; z1- and z2-setae transformed into claws, z3-seta untransformed; G1- and G3-claws reduced and short, G2-claw long (Fig. 11B). Md-palp (Fig. 11D) with 3+2 setae in bunch, gamma-seta pappose. Prehensile palps (Fig. 11E, F) with especially long, thin, and curved fingers. L6 (Fig. 11H) with basal seta reaching far beyond basal segment. L7 (Fig. 11G) clearly 5-segmented, penultimate segment only partly subdivided; basal segment with d1- and dp-setae; no e- or f-setae, g-seta long; terminal segment with setae h2- and h3- long and h1-seta shorter. UR (Fig. 11I) with curved anterior margin and short posterior seta. Hemipenis (Fig. 11J, 15F) with rounded and not projecting a-lobe; M-peace rounded distally, ejaculatory tube with pointed distal end. Zenker organ (Fig. 15F) with 5+2 whorls of spines; anterior cap hemispherical, with strongly sclerified margin, lattice elaborate, on anterior side adjoined by longer spines. Eyes large and dark.

#### Genus Baicalocandona

Taxon classificationAnimaliaPodocopidaCandonidae

Mazepova, 1976

##### Type species.

*Baicalocandona bivia* Mazepova, 1976

##### Amended diagnosis.

Shell shape (always) trapezoidal, surface ornamented in most species, at least in some parts. A1 7, 6 or 5-segmented. Male A2 with t-setae transformed into sensory setae, z-setae transformed into claws. Female A2 with G2-claw as long as G1 or G3. Exopod of A2 consisting of small plate and three setae of which one long. Male prehensile palps asymmetrical and both with hook-like fingers, but right palp with shorter, stockier and considerably less hook-like finger. L6 with basal seta and with one seta on each endopodal segment, except last, which carries two setae and one claw. L7 with only d1- and dp-seta on basal segment, e- and f-setae missing, g-seta long; terminal segment with short h1-seta and h2- and h3-setae equally long; penultimate segment fused without any notable subdivision. UR with both claws and setae present. Zenker organ with 4+2 whorls of spines. The anterior part (cap) more hemispherical and margin not sclerotized, lattice-like structure not well-developed; cap also with long radiating spine-like projections. Hemipenis with relatively large a-lobe not projecting laterally. M-peace terminally foot-like; ejaculatory process (bursa copulatrix) not terminally pointed, and with broad, rounded, finger-like extension; this process also with lateral thorn-like ornamented part.

##### Remarks.

*Baicalocandona* at the moment includes 11 species and 11 subspecies. According to the diagnosis (Mazepova 1976, 1990), all species have a trapezoidal valve shape, males have sexual bristles on the second antenna, and the Zenker organ bears six whorls of spines. One species we redescribe below falls within this diagnosis, although it was originally described in *Candona*. We also noted some other morphological characters that improve the genus diagnosis, such as a very short finger on the right prehensile palp, a short basal seta (d1) on the walking leg, undivided penultimate segment of the cleaning leg, a foot-like shape of the M-peace of hemipenis, and ejaculatory process finger like and pronounced. Females also seem to have a long G2-claw on the second antenna, and Zenker organ has long spine-like projections on anterior end. Based on the redescription of two *Pseudocandona* species, *P. ceratina* and *P. gajewskaye*, only the absence of the male sexual bristles on the second antenna separates this genus from *Baicalocandona*. The number of A1 segments, as well as the number of setae on the second segment of the Md-palp seems to be variable. *Baicalocandona* is very similar to the European subterranean genus, *Schellencandona*, both in the shell shape and morphology of the hemipenis.

#### Baicalocandona rupestris disona

Taxon classificationAnimaliaPodocopidaCandonidae

(Mazepova, 1990) comb. n.

**Figure 12.**
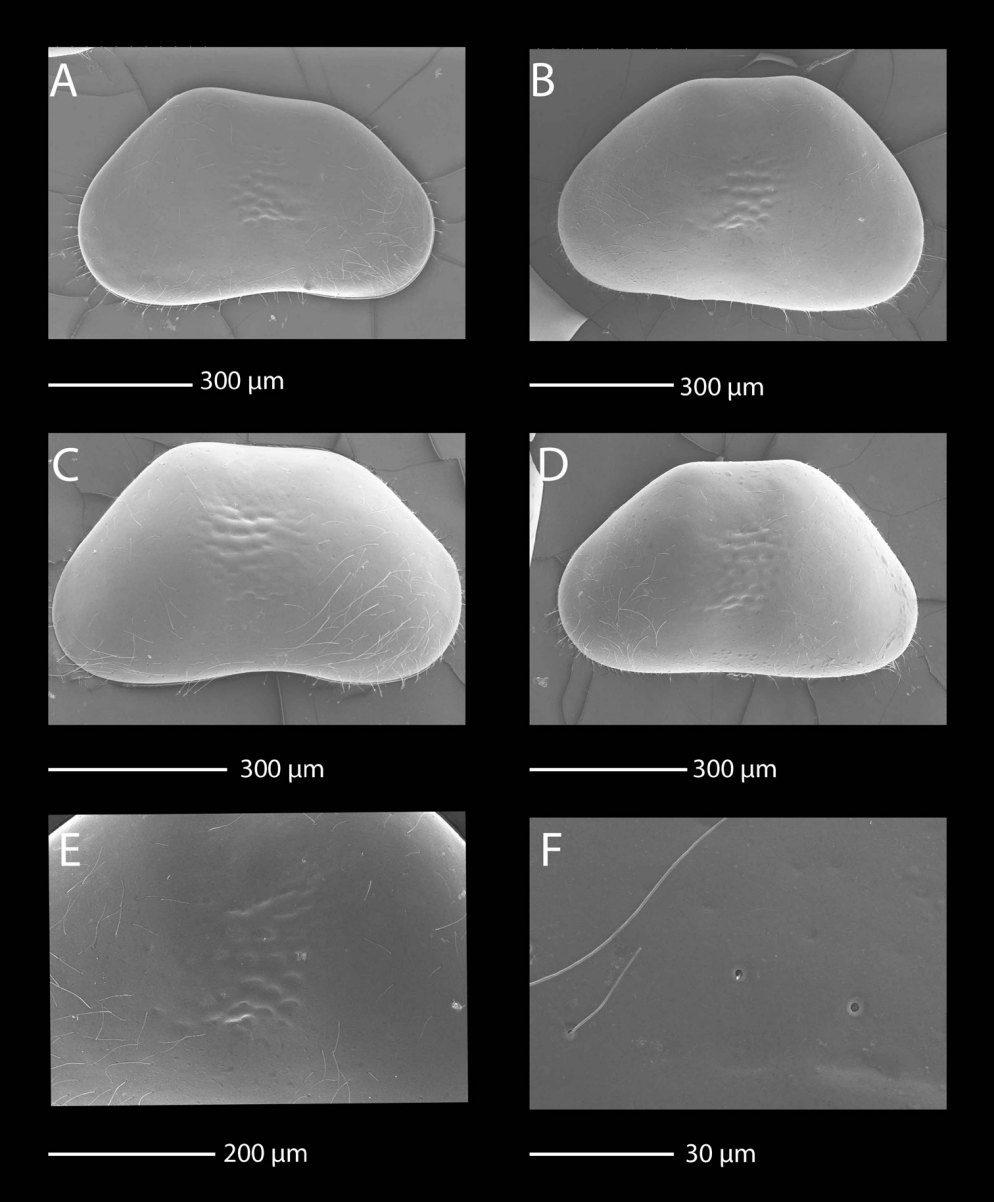
SEM photographs of *Baicalocandona rupestris disona* (Mazepova, 1990). **A, B, E, F** male **C, D** female **A** RV, lateral view **B** LV, lateral view **C** RV, lateral view **D** LV, lateral view **E** details of the fine surface ornamentation **F** details of shell surface pores and sensilla.

**Figure 13.**
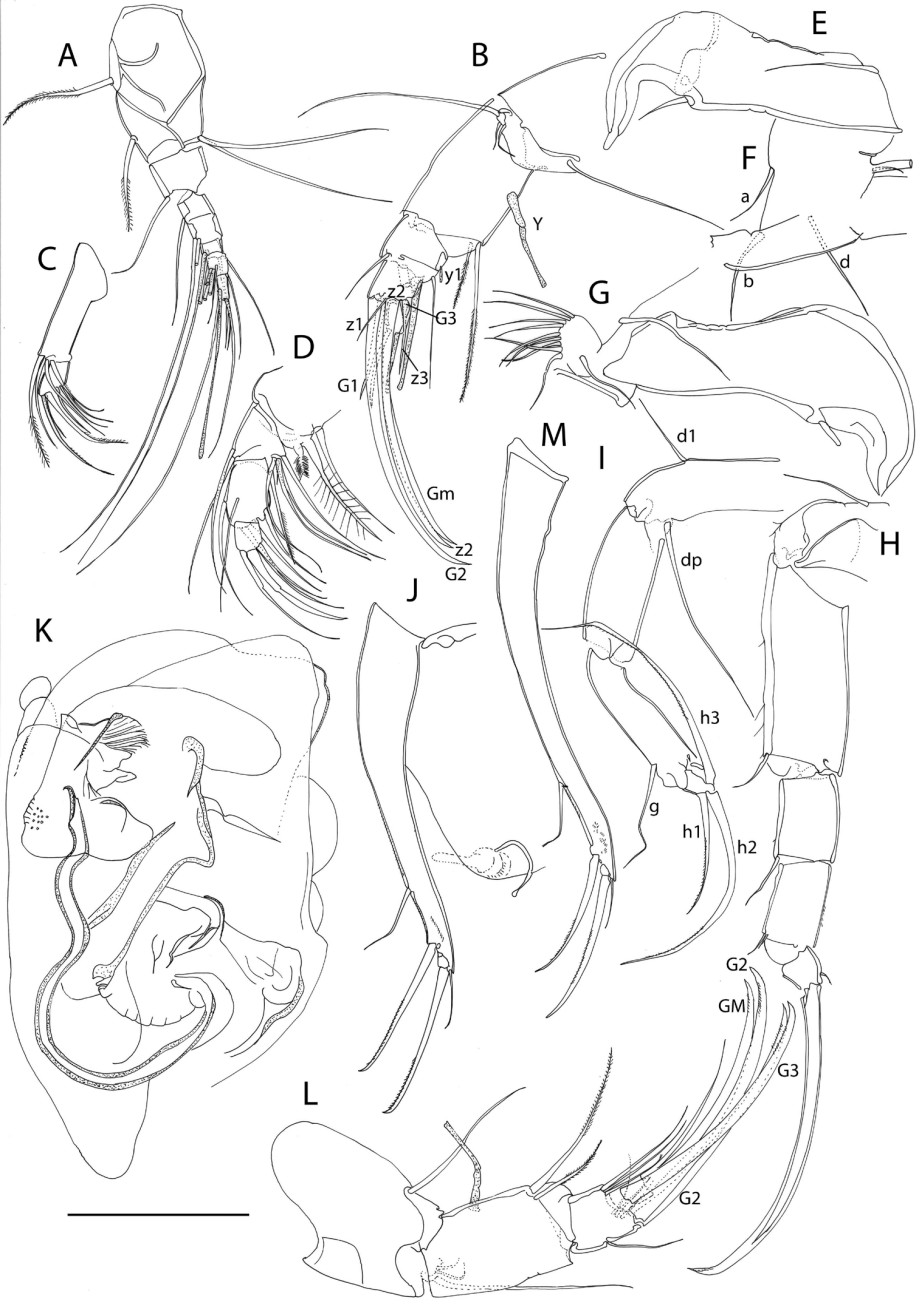
Line drawings of *Baicalocandona rupestris disona* (Mazepova, 1990) **A-E, G, H, I, K, M** male **F, J, L** female **A** A1 **B** A2 **C** Mxl-palp **D** Md-palp **E, G** prehensile palps **F** details of the L5 setae **H** L6 **I** L7 **J, M** UR **K** hemipenis.

**Figure 14.**
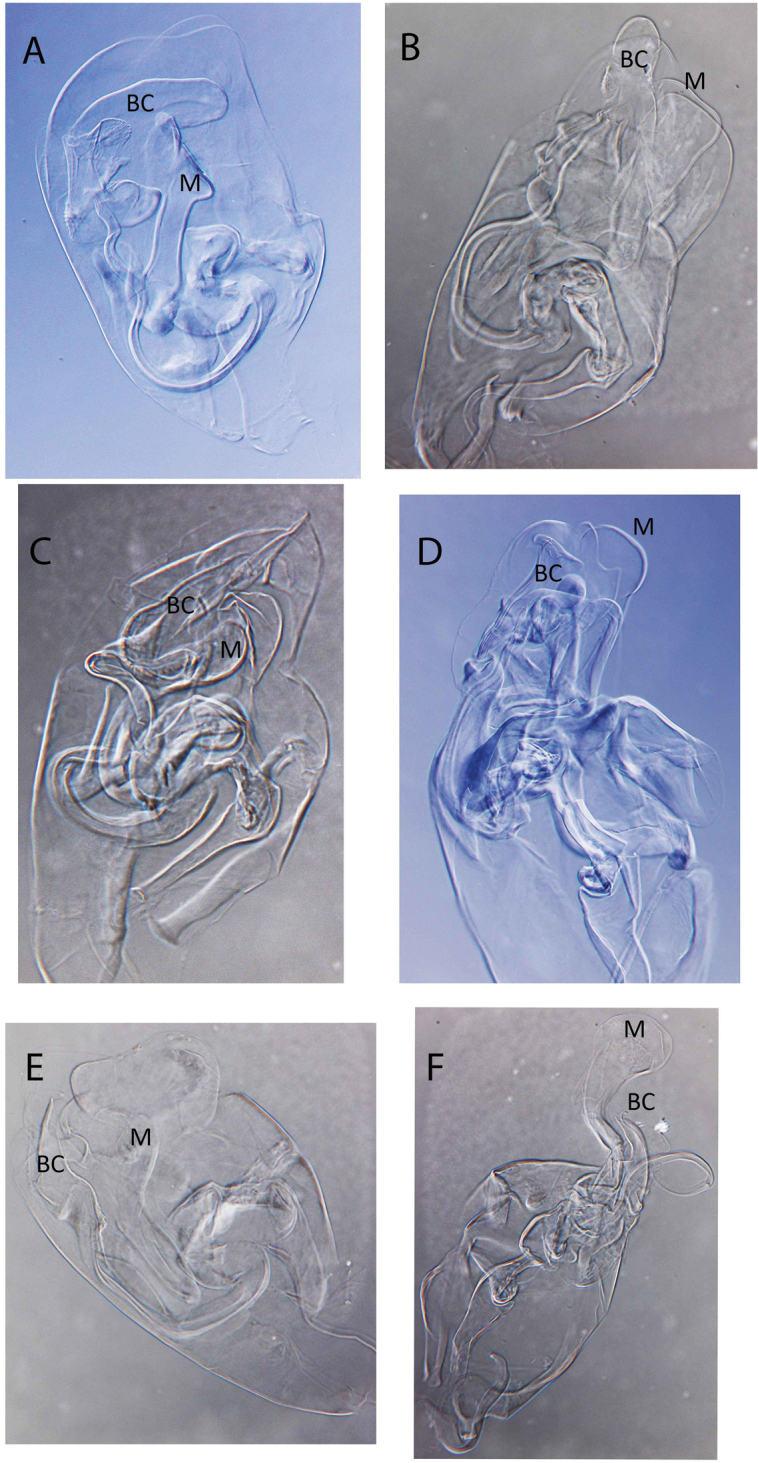
Light photographs of hemipenis. **A** *Baicalocandona rupestris disona* **B** *Baicalocandona* sp. **C** *Mazepovacandona navitarum* **D** *Mazepovacandona directa* **E** *Mazepovacandona orbiculata* **F** *Mazepovacandona spicata*. Not to scale.

**Figure 15.**
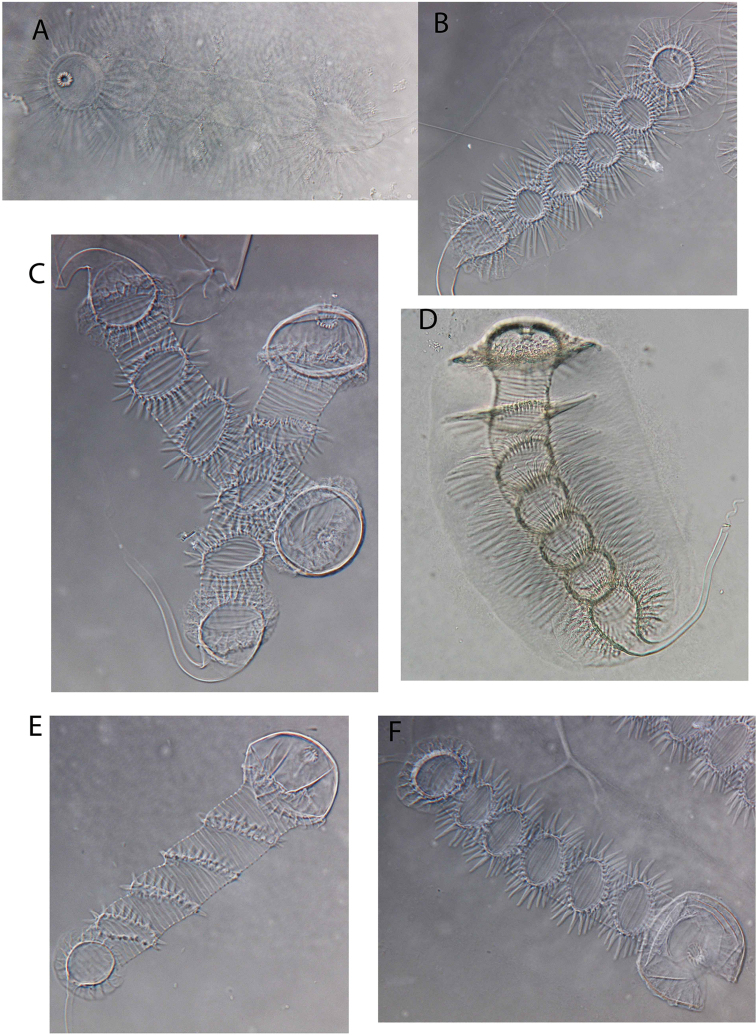
Light photographs of Zenker organ. **A** *Baicalocandona rupestris disona* **B** *Baicalocandona* sp. **C** *Mazepovacandona navitarum* **D** *Mazepovacandona directa* **E** *Mazepovacandona orbiculata* **F** *Mazepovacandona spicata*. Not to scale.

Figs 12 , 13 , 14A , 15A

Candona rupestris dissona subsp. n. – Mazepova (1990): p. 152, fig. 56B, 57B.

##### Material examined.

Soft parts of one male and one female used for DNA extraction, after that each dissected and mounted onto one glass slides, their shells kept on one SEM stub each, 40 juveniles kept in 95% alcohol, all collected from 12–15 m depth by SCUBA diving off Listvyanka, 51°51'51.3"N, 104°50'37.8"E, 12 September 2015, collectors: Igor Khanaev and Ivan Nebesnykh.

##### Short redescription.

Almost no sexual dimorphism in shell shape in lateral view (Fig. 12A–D). Both LV and RV trapezoidal, dorsal margin straight in middle and rounded/inclined towards anterior and posterior ends. Posterior and anterior ends narrower and anterior slightly wider than posterior end. Surface mostly smooth and ornamented with few shallow pits only centrally. Surface cuticular pores simple, without prominent lip. Surface sensory setae long (Fig. 12E, F). Length around 0.7 mm.

A1 7-segmented (Fig. 13A). Male A2 with subdivided penultimate segment and t2- and t3-setae transformed into sexual bristles; only z2-setae transformed into claws, z1, and z3-seta untransformed; G1- and G3-claws reduced and short, G2-claw long (Fig. 13B). Female A2 (Fig. 13M) with all three untransformed z-seta; G2 claw as long as all other claws. Md-palp (Fig. 13D) with 4+2 setae on inner side, gamma seta not plumose. Mxl-palp (Fig. 14C) with rectangular terminal segment. Prehensile palps (Fig. 13E, G) stocky, right one with very strong finger but not hook-like. L6 (Fig. 13H) with short basal seta. L7 (Fig. 13I) 4-segmented; basal segment with d1- and dp-setae; no e- or f-setae, g-seta long; terminal segment with setae h2- and h3- long and h1-seta shorter. UR in both sexes (Figs 13J, K) very similar, thin, and curved, with short posterior seta and thin, subequal claws. Hemipenis (Figs 13L, 14A) with large a-lobe but not laterally projecting, M-peace distally clearly foot-like, ejaculatory tube with large, finger-like distal end and with ornamented lateral plate. Female genital field (Fig. 13K) rounded. Zenker organ with 4+2 whorls of spines (Fig. 15A); anterior cap with thin margins and with long spine-like projections, and lattice not so well-pronounced. Eyes dark.

#### Baicalocandona

Taxon classificationAnimaliaPodocopidaCandonidae

sp.

Figs 14B , 15B

##### Material examined.

Soft parts of one male used for DNA extraction, after that dissected and mounted onto one glass slide, shell broken during dissection, collected from 12–15 m depth by SCUBA diving off Listvyanka, 51°51'51.3"N, 104°50'37.8"E, 12 September 2015, collectors: Igor Khanaev and Ivan Nebesnykh.

##### Short description.

A1 6-segmented. Male A2 with subdivided penultimate segment and t2- and t3-setae transformed into sexual bristles; both z1- z2-setae transformed onto claws; G1- and G3-claws reduced and short, G2-claw long. Md-palp with 4+2 setae on the inner side, gamma seta not plumose. Prehensile palps stocky and right one with a very strong finger but not hook-like. Hemipenis (Fig. 14B); with large a-lobe but not laterally projecting, M-peace distally clearly foot-like, ejaculatory tube with large, finger-like distal end and with ornamented lateral plate. Zenker organ with 4+2 whorls of spines (Fig. 15B); anterior cap with thin margins and with long spine-like projections, and lattice not so well-pronounced.

### Molecular analysis

BLAST analyses of the GenBank database revealed that the obtained sequences were ostracod in origin and not contaminants. No stop codons were detected in the *COI* sequences. The *COI* alignment was 672 base pairs long, and included four species each with one sequence. The concatenated dataset was 3302 base pairs long, and it included 50 sequences belonging to 39 species. Of the individual alignments, 18S dataset was the longest (1042 positions) and also included 50 terminals. The alignment of 16S was the shortest (554 base pairs), and had only 21 species. After the exclusion of ambiguous blocks, 28S alignments varied from 660 base pairs (em fragment) to 455 base pairs (df fragment). The vx primer pair was the most successful in amplifying the region, while df fragment was very difficult to amplify and only 34 sequences were analyzed. The amplification by em primer pair was relatively successful, but this was the most difficult dataset to aligned due to the long expansion segments present in several species. Although initially this alignment was very long (1521 base pairs), after the Gblock analysis (Castresana 2000) it was truncated substantially.

GTR (Rodríguez et al. 1990) with unequal rates among sites, with gamma distribution and invariable site (GTR + G + I) for 18S, 16S, 28S (df and vx fragments), but without invariable sites for 28S em fragment, was chosen as the best fit evolutionary model.

**Figure 16.**
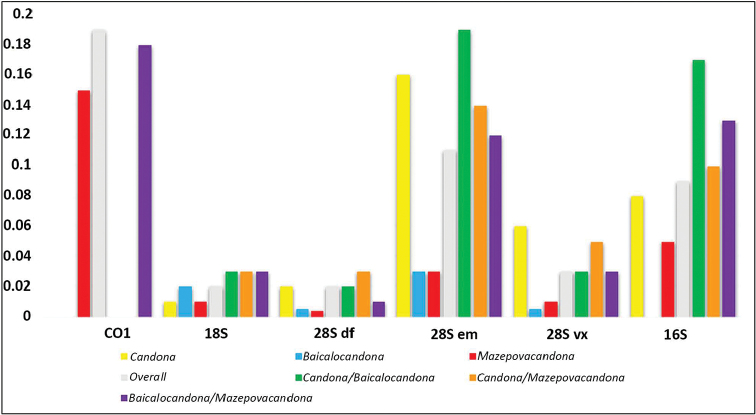
Pairwise *p*-distances for individual datasets.

The results of p-distance analysis are shown in Fig. 16, which illustrates overall, within, and between genera distances for each analyzed gene. Here we presented only distances relevant to the Baikal candonids and their closest relative, *Candona*. In the calculations, we reated sequences belonging to the two unidentified *Pseudocandona* species as *Baicalocandona* because they nest within *Baicalocandona* species on the phylogenetic tree (see below) and adding another genus name on the Figure 16 would introduce unnecessary confusion. All numerical data related to the p-distances are provided in the Supplements 2–7. The amplification of the *COI* region was not very successful, and we obtained a single sequence of *Baicalocandona* and three of the *Mazepovacandona* species. Nevertheless, it shows that the distances within *Mazepovacandona* are smaller than between it and *Baicalocandona*. The largest *COI* distance was between *B. rupestris* and *M. directa* (23%). Of the three 28S fragments, the em fragment was the most variable, exceeding even the variability of 16S. *Candona* and *Baicalocandona* diverged the most, with almost 20% differences. Divergence between *Candona* and *Mazepovacandona* was about 15%, and *Baicalocandona* and *Mazepovacandona* about 12%. *Candona* also had a very large within group variability of the em fragment (16%), which is very unusual and maybe because some of the sequences had extensive regions of nucleotide insertions (indels). The em fragment's variability is followed by fragment vx, where again *Candona* had the largest within group variability in comparison to the other two genera. The df fragment was very conservative, with maximum 4% differences found between *Candona* and *Mazepovacandona*. This fragment's variability was almost the same as the variability recorded for 18S. Of the three genera, *Baicalocandona* had the highest within group distance of 18S sequences, however only about 3%, while in the other two genera the distances were less than 2%. Between genera distances were almost identical, equaling 4%. *Candona* and *Baicalocandona* had the highest p-distance between their 16S sequences (18%). The distance between the latter genus and *Mazepovacandona* was around 14%. *Candona* and *Mazepovacandona* had only 10% differences between their 16S sequences. Except for the 16S, all other examined sequence distances were smaller between the two Baikal genera than between any of them and *Candona*. On the other hand, distances were sometimes higher between *Candona* and *Baicalocandona* and sometimes between *Candona* and *Mazepovacandona*, depending on the marker.

**Figure 17.**
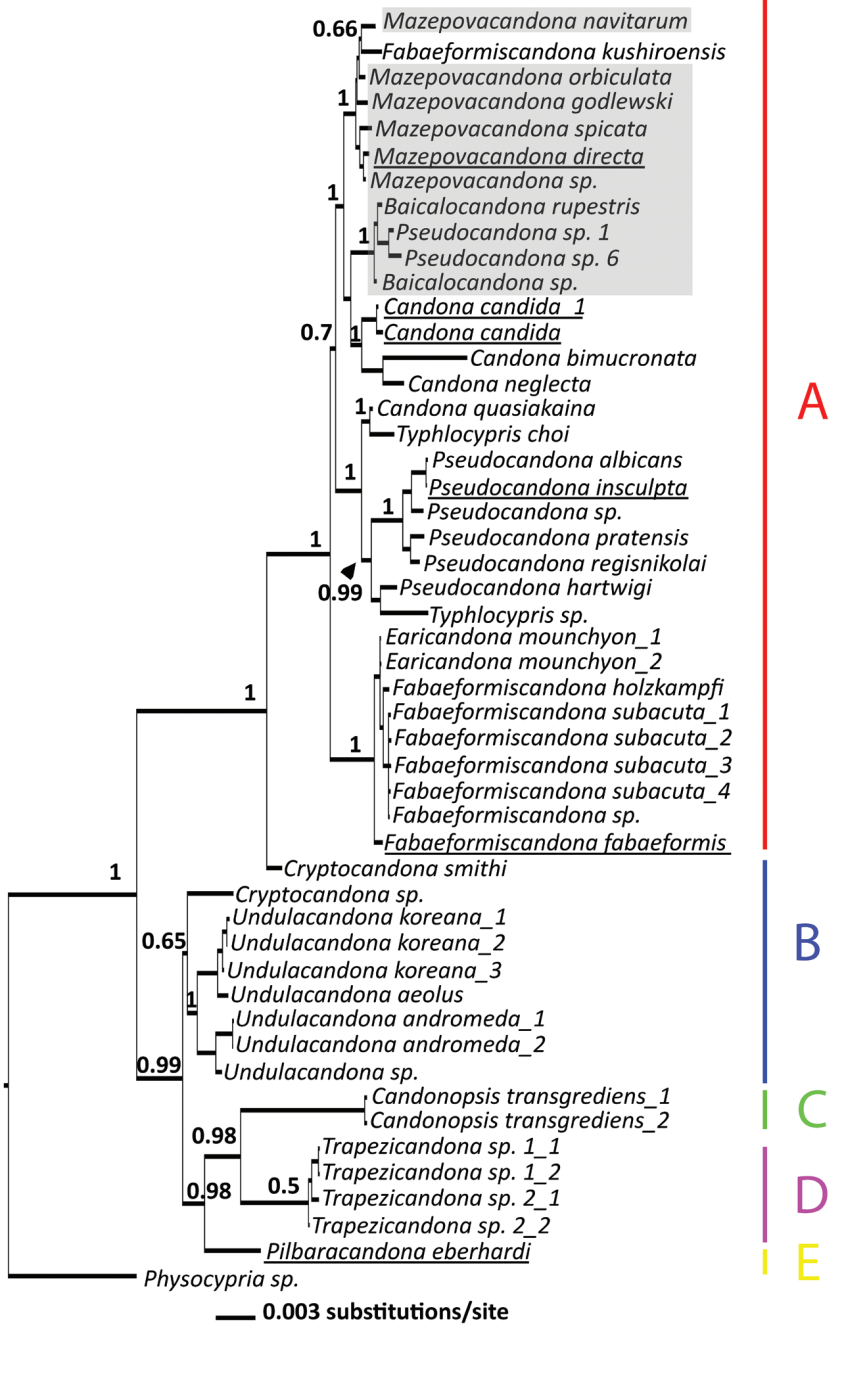
50% majority rule consensus tree of the family Candonidae constructed from the concatenated dataset of two nuclear (18S & 28S) and one mitochondrial (16S) markers. Numbers on the branches represent Bayesian posterior probabilities. Underlined taxa represent type species. Grey shaded taxa are Lake Baikal species. Tree rooted with *Physocypria* sp. Tribes are labeled with letters: **A** Candonini **B** Cryptocandonini **C** Candonopsini **D** Trapezicandonini **E** Humphreyscandonini.

After two million generation runs in MrBayes, the final standard deviation of split frequencies fell below 0.01 (it was around 0.003) and the potential scale reduction factor was ~1.0 for all parameters, suggesting that convergence had been reached. All resulting consensus trees were rooted with the outgroup, *Physocypria* sp. Fig. 17 illustrates the 50% consensus tree resulting from the analysis of the concatenated dataset. On this tree Candonidae is strongly supported as a monophyletic group. The Candonidae clade can be broadly divided into two subclades, both with high posterior probability values: one containing 15 sequences equating to nine species, and the other which incorporates 34 sequences belonging to 28 species. The former clade contained four Candonidae tribes, proposed by Karanovic (2007): Cryptocandonini, Candonopsini, Trapezicandonini, and Humphreyscandonini. Candonopsini was a sister taxon to Trapezicandonini, while Humphreyscandonini was the sister taxon to these two. These relationships received a relatively high posterior probability support, while the clade consisting of the two Cryptocandonini genera (*Cryptocandona* and *Undulacandona*) did not have high posterior probability.

The larger clade on the tree was composed of two tribes. All except *Cryptocandona smithi* Karanovic & Lee, 2012 belong to the largest Candonidae tribe, Candonini. Candonini can be broadly divided into three clades, all with maximum posterior probabilities. Ten Lake Baikal candonids did not form a monophyletic clade, but clustered with some non-Baikal species, in particular *Fabaeformiscandona kushiroensis*, *Candona candida*, *C. bimucronata*, and *C. neglecta*. *Fabaeformiscandona kushiroensis* is nested within the *Mazepovacandona* clade. The clade composed of the second Baikal lineage and three *Candona* species received a very low support (below 0.5 posterior probability). A clade composed of nine species belonging to *Candona*, *Pseudocandona*, and *Typhlocypris* was sister to the previous, mostly composed of Baikal candonids, but this association did not have high posterior probability (0.7). The last group on the tree, consisting of *Earicandona* and *Fabaeformiscandona*, was strongly supported and was sister to the previous two clades.

## Discussion

When defining Baikal genera, we were mostly lead by the results of the molecular phylogeny analysis, which indicated that the 10 Baikal species belong to only two lineages. However, the morphological diversity of Lake Baikal candonids is extraordinary, especially when compared with the candonid fauna from other parts of the world. In fact, when compared with the Holarctic candonid genera, each *Mazepovacandona* species redescribed in this paper has enough apomorphic characters (from the shell shape to the number of whorls on the Zenker organ) to be described in a separate genus. In addition, Mazepova's (1990) descriptions clearly show that each of the species redescribed here (but also many others) has one or more sister species in the lake. For example, *Candona humilis* Bronstein, 1939; *C. unguicaudata* Bronstein, 1930; *C. semilunaris dignitosa* Mazepova, 1990, and few other have a very similar carapace shape, hemipenis and prehensile palps to *M. directa*. Similarly, *C. muriformis* Mazepova, 1984; *C. unimodal* Mazepova, 1984; and *C. birsteini* Mazepova, 1990 have a lot of common morphological characters with *M. orbiculata*. On the other hand, representatives of *Baicalocandona* seem to be more morphologically uniform (starting with a trapezoidal shape of the shell) and this genus may even include a few Baikal *Pseudocandona* species. This large morphological and low molecular diversity of Lake Baikal Candonids is contributing to a long list of animal groups where morphological and molecular evolution have been uncoupled (Pisani et al. 2007; Renaud et al. 2007; Sotiaux et al. 2009; Poisot et al. 2011; Dávalos et al. 2012). It is interested to note that Schön and Martens (2012) recovered several distinct clades in the Lake Baikal cytheroid ostracods based on the *COI* sequences. Although, at the moment they all belong to the same genus, the authors suggest a taxonomic revision and more detail morphological studies.

Based on our phylogenetic tree, none of the Baikal species included in this study could be assigned to either *Candona* or *Pseudocandona*, as demonstrated by the position of the type species of these two genera (underlined species on the tree). Nevertheless, they are morphologically and genetically more closely related to *Candona* than to any other Candonidae genera included in this analysis. *Candona* is a polyphyletic taxon, which is illustrated by the fact that most (if not all) of the *Candona* species endemic to Baikal Lake should be excluded from it, and by the position of *C. quasiakaina* Karanovic & Lee, 2012 nested within the true *Pseudocandona/Typhlocypris* clade on the tree. *Fabaeformiscandona* is also a polyphyletic genus, which was already speculated many times (Karanovic 2006, 2007, 2013, etc.). The position of *Fabaeformiscandona kushiroensis* nested within the *Mazepovacandona* clade is an additional evidence. This Japanese species strongly differs from the typical *Fabaeformiscandona* species, and its affinity with *Mazepovacandona* can be seen in the morphology of the M-peace and ejaculatory tube of the hemipenis (see Hirutaand Hiruta 2015). There have been several attempts to revise *Candona* and *Fabaeformiscandona* which are the two largest Candonidae genera (Karanovic 2006), but there is still no consensus among the ostracodologists regarding the importance of many morphological characters (such as the shape of the shell, number of setae on the mandibular palp, morphology of the “gamma” seta on the same appendage, etc.). In the morphological cladistic analysis performed by Karanovic (2007) these characters were extremely homoplastic. Sexual characters (such as the morphology of the hemipenis) will probably prove to best reflect the generic groupings, and they should be built upon already existing morphological characterizations proposed by Petkovski (1960) and Danielopol (1969). The polyphyletic nature of *Typhlocypris* on the tree is partly a result of the nomenclature disagreements regarding the names *Typhlocypris* and *Pseudocandona* (Karanovic 2005; Namiotko et al. 2014). Morphological characters proposed by Namiotko et al. (2014) for *Typholcypris* s. str. seem to warrant future better systematics of both genera. The polyphyletic nature of *Cryptocandona* has been suggested by Karanovic and Lee (2012) based on peculiar morphological characters of two species found in East Asia and one from Sweden. The present molecular analysis as well as the one performed by Karanovic and Cho (2017) confirms this. Systematic revision of *Candona*, *Cryptocandona*, *Fabaeformiscandona*, *Pseudocandona*, and *Typhlocypris* is beyond the scope of the present paper. The position of *F. kushiroensis* on the tree suggests that its ancestors originated in the lake. There are many similar examples in other Baikal groups. Sculpin fishes have a high diversity in Baikal and one closely related species in Lake Michigan (see Sherbakov 1999); endemic Baikal mollusks have a relative in Mongolian lakes (Papusheva et al. 1997); and an amphipod species found in Finnish streams has closest relatives in Baikal (Vainola et al. 1995). Karanovic and Abe (2010) and Karanovic et al. (2013) attributed to ancient lakes a role of biodiversity pumps for subterranean habitats in addition to their role as refugia, because their deep and dark benthic environments provide ideal conditions for the evolution of subterranean adaptations. Morphological affinity between *Baicalocandona* and *Schellencandona*, which is distributed in subterranean waters of Europe, is one example supporting this hypothesis.

Molecular diversity of gene markers commonly used for resolving higher phylogenetic relationships (18S and 28S) is relatively small between Baikal candonids and their closest relatives, in comparison to other ostracod lineages. For example, in the family Cyprididae, distances between 18S sequences vary from 2% (within genus) to 11% (between genera) (Kong et al. 2014); while in Polycopidae the same marker has approximately 3% intragenic and 10% intergeneric variability (Tanaka et al. 2014; Karanovic et al. 2016). The distances between *COI* sequences of the four Baikal candonids are within the range of those observed for other ostracods and crustaceans in general (Lefébure et al. 2006; Schön and Martens 2012; Schön et al. 2015, 2017).

## Supplementary Material

XML Treatment for Mazepovacandona

XML Treatment for Mazepovacandona directa

XML Treatment for Mazepovacandona godlewski

XML Treatment for Mazepovacandona navitarum

XML Treatment for Mazepovacandona orbiculata

XML Treatment for Mazepovacandona spicata

XML Treatment for Baicalocandona

XML Treatment for Baicalocandona rupestris disona

XML Treatment for Baicalocandona

## Supplementary materials

Supplementary material 1 Locality data and BenBank Accession Numbers Data type: molecular data Explanation note: Species in bold are our sequences, regular font species were downloaded from the GenBank. This dataset is made available under the Open Database License (http://opendatacommons.org/licenses/odbl/1.0/). The Open Database License (ODbL) is a license agreement intended to allow users to freely share, modify, and use this Dataset while maintaining this same freedom for others, provided that the original source and author(s) are credited.Ivana Karanovic, Tatiana Y. Sitnikova

Supplementary material 2 p-distances between 18S rRNA sequences Data type: statistical data Explanation note: data were used for the Figure 16. This dataset is made available under the Open Database License (http://opendatacommons.org/licenses/odbl/1.0/). The Open Database License (ODbL) is a license agreement intended to allow users to freely share, modify, and use this Dataset while maintaining this same freedom for others, provided that the original source and author(s) are credited.Ivana Karanovic, Tatiana Y. Sitnikova

Supplementary material 3 p-distances between 28S rRNA sequences, df region Data type: statistical data Explanation note: data were used for the Figure 16. This dataset is made available under the Open Database License (http://opendatacommons.org/licenses/odbl/1.0/). The Open Database License (ODbL) is a license agreement intended to allow users to freely share, modify, and use this Dataset while maintaining this same freedom for others, provided that the original source and author(s) are credited.Ivana Karanovic, Tatiana Y. Sitnikova

Supplementary material 4 p-distances between 28S rRNA sequences, em region Data type: statistical data Explanation note: data were used for the Figure 16. This dataset is made available under the Open Database License (http://opendatacommons.org/licenses/odbl/1.0/). The Open Database License (ODbL) is a license agreement intended to allow users to freely share, modify, and use this Dataset while maintaining this same freedom for others, provided that the original source and author(s) are credited.Ivana Karanovic, Tatiana Y. Sitnikova

Supplementary material 5 p-distances between 28S rRNA sequences, vx region Data type: statistical data Explanation note: data were used for the Figure 16. This dataset is made available under the Open Database License (http://opendatacommons.org/licenses/odbl/1.0/). The Open Database License (ODbL) is a license agreement intended to allow users to freely share, modify, and use this Dataset while maintaining this same freedom for others, provided that the original source and author(s) are credited.Ivana Karanovic, Tatiana Y. Sitnikova

Supplementary material 6 p-distances between 16S rRNA sequences Data type: statistical data Explanation note: data were used for the Figure 16. This dataset is made available under the Open Database License (http://opendatacommons.org/licenses/odbl/1.0/). The Open Database License (ODbL) is a license agreement intended to allow users to freely share, modify, and use this Dataset while maintaining this same freedom for others, provided that the original source and author(s) are credited.Ivana Karanovic, Tatiana Y. Sitnikova

Supplementary material 7 p-distances between *COI* sequences Data type: statistical data Explanation note: data were used for the Figure 16. This dataset is made available under the Open Database License (http://opendatacommons.org/licenses/odbl/1.0/). The Open Database License (ODbL) is a license agreement intended to allow users to freely share, modify, and use this Dataset while maintaining this same freedom for others, provided that the original source and author(s) are credited.Ivana Karanovic, Tatiana Y. Sitnikova
